# Rituximab versus tocilizumab in rheumatoid arthritis: synovial biopsy-based biomarker analysis of the phase 4 R4RA randomized trial

**DOI:** 10.1038/s41591-022-01789-0

**Published:** 2022-05-19

**Authors:** Felice Rivellese, Anna E. A. Surace, Katriona Goldmann, Elisabetta Sciacca, Cankut Çubuk, Giovanni Giorli, Christopher R. John, Alessandra Nerviani, Liliane Fossati-Jimack, Georgina Thorborn, Manzoor Ahmed, Edoardo Prediletto, Sarah E. Church, Briana M. Hudson, Sarah E. Warren, Paul M. McKeigue, Frances Humby, Michele Bombardieri, Michael R. Barnes, Myles J. Lewis, Costantino Pitzalis, the R4RA collaborative group, Felice Rivellese, Giovanni Giorli, Alessandra Nerviani, Liliane Fossati-Jimack, Georgina Thorborn, Frances Humby, Michele Bombardieri, Myles J. Lewis, Patrick Durez, Maya H. Buch, Hasan Rizvi, Arti Mahto, Carlomaurizio Montecucco, Bernard Lauwerys, Nora Ng, Pauline Ho, Vasco C. Romão, João Eurico Cabral da Fonseca, Patrick Verschueren, Stephen Kelly, Pier Paolo Sainaghi, Nagui Gendi, Bhaskar Dasgupta, Alberto Cauli, Piero Reynolds, Juan D. Cañete, Julio Ramirez, Raquel Celis, Robert Moots, Peter C. Taylor, Chris J. Edwards, John Isaacs, Peter Sasieni, Ernest Choy, Charlotte Thompson, Serena Bugatti, Mattia Bellan, Mattia Congia, Christopher Holroyd, Arthur Pratt, Laura White, Louise Warren, Joanna Peel, Rebecca Hands, Gaye Hadfield, Costantino Pitzalis

**Affiliations:** 1 Centre for Experimental Medicine and Rheumatology, William Harvey Research Institute, Barts and The London School of Medicine and Dentistry, Queen Mary University of London, London, UK; 2 Centre for Translational Bioinformatics, William Harvey Research Institute, Barts and the London School of Medicine and Dentistry, Queen Mary University of London, London, UK; 3 NanoString Technologies Inc., Seattle, WA USA; 4 Usher Institute, College of Medicine and Veterinary Medicine, University of Edinburgh, Edinburgh, UK; 5 Department of Rheumatology, Cliniques Universitaires Saint-Luc, Brussels, Belgium; 6 Institute of Experimental and Clinical Research, Université catholique de Louvain, Brussels, Belgium; 7 Centre for Musculoskeletal Research, Division of Musculoskeletal & Dermatological Sciences, The University of Manchester, Manchester, UK; 8 NIHR Manchester Biomedical Research Centre, Manchester, UK; 9 Department of Cellular Pathology, Barts Health NHS Trust, London, UK; 10 Institute of Health Sciences Education, Queen Mary University of London, London, UK; 11 Department of Rheumatology, Kings College Hospital NHS Foundation Trust, London, UK; 12 Department of Rheumatology, Fondazione I.R.C.C.S. Policlinico San Matteo, University of Pavia, Pavia, Italy; 13 Rheumatology Department, Guy’s and St Thomas’ NHS Foundation Trust, London, UK; 14 The Kellgren Centre for Rheumatology, Manchester Royal Infirmary, Manchester University NHS Foundation Trust, Manchester, UK; 15 Rheumatology Department, Hospital De Santa Maria Centro Hospitalar Universitário Lisboa Norte, Lisbon, Portugal; 16 Rheumatology Research Unit, Instituto de Medicina Molecular João Lobo Antunes, Faculdade de Medicina, Universidade de Lisboa, Lisbon, Portugal; 17 Skeletal Biology and Engineering Research Centre, Department of Development and Regeneration, KU Leuven, Leuven, Belgium; 18 Division of Rheumatology, University Hospital Leuven, Leuven, Belgium; 19 Department of Rheumatology, University of Eastern Piedmont and Maggiore della Carita Hospital, Novara, Italy; 20 Rheumatology Department, Basildon Hospital, Basildon, UK; 21 Rheumatology Department, Mid & South Essex University Hospitals NHS Foundation Trust, Southend University Hospital, Westcliff-on-Sea, UK; 22 Rheumatology Unit, Department of Medicine and Public Health, AOU and University of Cagliari, Monserrato, Italy; 23 Department of Rheumatology, Homerton University Hospital, London, UK; 24 Rheumatology Department, Hospital Clínic de Barcelona, Barcelona, Spain; 25 Institut d’Investigacions Biomèdiques August Pí I Sunyer, Barcelona, Spain; 26 Academic Rheumatology Unit, Aintree University Hospital, Liverpool, UK; 27 Faculty of Health, Social Care and Medicine, Edge Hill University, Ormskirk, UK; 28 Nuffield Department of Orthopaedics, Rheumatology and Musculoskeletal Sciences, Botnar Research Centre, University of Oxford, Oxford, UK; 29 NIHR Clinical Research Facility, University Hospital Southampton, Southampton, UK; 30 Faculty of Medicine, University of Southampton, Southampton, UK; 31 Translational and Clinical Research Institute, Newcastle University, Newcastle upon Tyne, UK; 32 Musculoskeletal Unit, Newcastle upon Tyne hospitals NHS Foundation Trust, Newcastle upon Tyne, UK; 33 King’s Clinical Trials Unit, Kings College London, London, UK; 34 CREATE Centre, Cardiff University, Cardiff, UK; 35 Department of Rheumatology, University Hospital of Wales, Cardiff, UK; 36 Brighton and Sussex Medical School, University of Sussex, Brighton, UK; 37 Southampton General Hospital, University Hospital Southampton NHS Foundation Trust, Southampton, UK; 38 Cancer Research UK & UCL Cancer Trials Centre, University College London, London, UK

**Keywords:** Predictive markers, Randomized controlled trials, Predictive medicine, Rheumatoid arthritis, Machine learning

## Abstract

Patients with rheumatoid arthritis (RA) receive highly targeted biologic therapies without previous knowledge of target expression levels in the diseased tissue. Approximately 40% of patients do not respond to individual biologic therapies and 5–20% are refractory to all. In a biopsy-based, precision-medicine, randomized clinical trial in RA (R4RA; *n* = 164), patients with low/absent synovial B cell molecular signature had a lower response to rituximab (anti-CD20 monoclonal antibody) compared with that to tocilizumab (anti-IL6R monoclonal antibody) although the exact mechanisms of response/nonresponse remain to be established. Here, in-depth histological/molecular analyses of R4RA synovial biopsies identify humoral immune response gene signatures associated with response to rituximab and tocilizumab, and a stromal/fibroblast signature in patients refractory to all medications. Post-treatment changes in synovial gene expression and cell infiltration highlighted divergent effects of rituximab and tocilizumab relating to differing response/nonresponse mechanisms. Using ten-by-tenfold nested cross-validation, we developed machine learning algorithms predictive of response to rituximab (area under the curve (AUC) = 0.74), tocilizumab (AUC = 0.68) and, notably, multidrug resistance (AUC = 0.69). This study supports the notion that disease endotypes, driven by diverse molecular pathology pathways in the diseased tissue, determine diverse clinical and treatment–response phenotypes. It also highlights the importance of integration of molecular pathology signatures into clinical algorithms to optimize the future use of existing medications and inform the development of new drugs for refractory patients.

## Abstract (web summary)

Biomarker analysis of the phase 4 R4RA trial identifies pretreatment synovial biopsy features selectively associated with response to rituximab or tocilizumab, and leads to the development of models that might predict treatment benefit in patients with rheumatoid arthritis

## Main

Treatment of RA has been transformed by the introduction of therapeutics directed against soluble mediators (for example, tumor necrosis factor (TNF) inhibitors and IL6R blockers), immune cells (for example, B cells) and intracellular signaling pathways (Janus kinase inhibitors)^1^. However, approximately 40% of patients do not respond to individual agents while 5–20% are resistant to all current medications^2^. The mechanisms of nonresponse are largely unknown and, unlike in other medical fields such as cancer where molecular pathology guides the use of targeted therapies^3,4^, biomarkers able to predict response to specific agents in RA are still lacking^5^. Because RA is highly heterogeneous, it is plausible that different pathways are active in individual patients^6^. For example, because approximately 50% of patients with RA display low/absent CD20^+^ B cells in diseased joint tissue (synovium)^7^, the target for the anti-CD20 rituximab monoclonal antibody, it has been postulated that the level of synovial B cells/B cell-related pathways would influence treatment response to rituximab. However, results from small observational studies provide inconsistent and inconclusive results^8^.

To address this hypothesis we carried out a biopsy-driven, randomized clinical trial in RA (R4RA)^9^ in which TNF-inhibitor-inadequate responders were randomized to either rituximab (anti-CD20 monoclonal antibody) or tocilizumab (anti-IL6R monoclonal antibody) after stratification according to synovial B cell signatures. The trial results demonstrated that only 12% of patients with a low synovial B cell molecular signature responded to rituximab while 50% responded to tocilizumab. In contrast, in patients with high synovial B cell lineage signature, the two drugs appeared comparably effective.

Here, we investigated the mechanisms of response and nonresponse to these two targeted biologics through deep histopathological and molecular (RNA-sequencing (RNA-Seq)) characterization of synovial tissue at baseline, and longitudinally in post-treatment biopsies at 16 weeks. We identified specific signatures associated with therapeutic response and developed machine learning classifiers to predict treatment response. Additionally, we provide insights into the cellular and molecular pathways underpinning multidrug resistance defining a refractory phenotype, characterized by a stromal/fibroblast signature. Finally, digital spatial profiling of synovial biopsies highlighted differences in gene expression in specific synovial regions with relevance to disease pathogenesis and treatment response.

## Results

### Histological and in silico cell lineages correlate with drug response

**Fig. 1:**
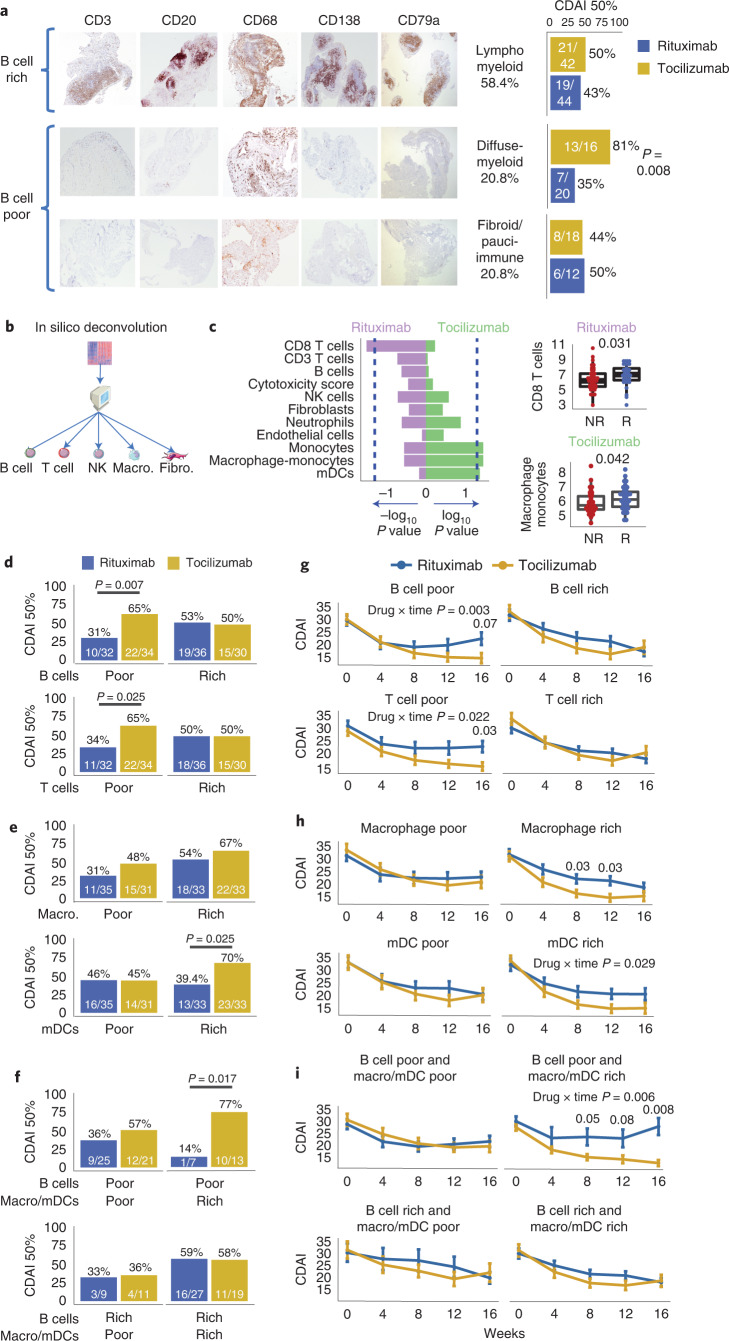
Synovial histological markers at baseline associate with response to rituximab and tocilizumab. **a**, Classification into synovial pathotypes according to semiquantitative scores for CD3^+^ T cells, CD20^+^ B cells, CD68^+^ macrophages and CD138^+^ plasma cells, with representative examples from patients classified as lymphomyeloid (CD20 ≥ 2 and/or CD138≥2), diffuse-myeloid (CD68SL≥2, and CD20/CD138<2) or fibroid/pauci-immune (CD68SL/CD20/CD138<2). Right, 16-week CDAI 50% response in patients stratified by pathotype (*n* = 152). Bar plots showing the proportion of CDAI 50% responders for rituximab (in blue) and tocilizumab (in yellow) within each pathotype, with corresponding exact numbers. Fisher's test, exact *P* values for *P* < 0.05. **b**, Approach to in silico deconvolution of synovial tissue using MCP-counter. **c**, MCP-counter scores for each cell type compared among CDAI 50% responders (R) and nonresponders (NR). Bar plots indicate nominal log_10_ *P* values for tocilizumab and –log_10_ *P* values for rituximab (two-sided Mann–Whitney test); dashed lines correspond to *P* = 0.05. Boxplots (right) show median and first and third quartiles, whiskers extending to the highest and lowest values. **d**–**f**, 16-week CDAI 50% response in patients stratified into B and T cell poor/rich (**d**) and macrophage/mDC poor/rich (**e**) according to median MCP-counter scores for individual cells (rich if above median, poor if below), or by combining B cell and macrophage/mDC scores from **d**,**e** (**f**). Exact *P* values shown when <0.05, two-sided Fisher's test comparing the proportions of responders to rituximab (in blue) and tocilizumab (in yellow). **g**–**i**, Longitudinal disease activity scores (CDAI), shown as mean ± s.d., for each month from baseline to 16 weeks for patients randomized to rituximab (in blue) or tocilizumab (in yellow) and classified as B and T cell poor/rich (**g**), macrophage/mDC poor/rich (**h**) and combined B cell/macrophage poor/rich (**i**). Comparison of CDAI between the two medications at individual time points by two-sided Mann–Whitney test, exact *P* values for <0.05 (adjustment for multiple comparisons by FDR). *P* values for the drug × time interaction term (two-way repeated-measures analysis of covariance) are shown when <0.05. **c**–**i**, *n* = 133 patients with baseline RNA-seq. NK, natural killer cells. mDC, myeloid dendritic cells.

**Extended Data Fig. 1:**
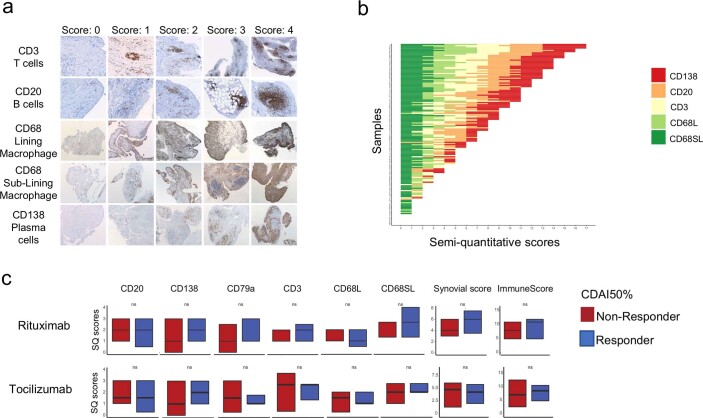
Histological analyses. **a**, Atlas of semi-quantitative synovial IHC scores for immune cells. **b**, Distribution of semiquantitative scores at baseline in all patients, individually shown in the y axis. The total on the x axis represents the sum of the individual scores (Immune score). **c**, Baseline semi-quantitative IHC scores, Krenn synovitis score (‘Synovial score’) and total Immune score in patients stratified according to 16 weeks CDAI50% response to rituximab (top) and tocilizumab (bottom). Two-sided Mann Whitney test. ns= p value >0.05. n = 161 patients. Boxplots showing median with first and third quartiles.

To assess the association of synovial immune cells with treatment response, we compared semiquantitative immunohistochemistry (IHC) scores (Extended Data Fig. 1a,b) in pretreatment synovial biopsies of responders (*n* = 28 for rituximab, *n* = 37 for tocilizumab) and nonresponders (*n* = 54 and *n* = 42, respectively), showing no differences (Extended Data Fig. 1c). However, when patients were stratified according to previously described^6,7^ synovial histological patterns, also known as pathotypes (Fig. 1a), patients with a diffuse-myeloid pathotype, i.e. with myeloid lineage predominance but low in B/plasma cells, displayed a significantly higher response to tocilizumab (13/16, 81%) versus rituximab (7/20, 35%) (*P* = 0.008, odds ratio (OR) = 7.53, 95% confidence interval (CI) 1.4–55.7). In contrast, similar response rates between treatments were observed in patients with a lymphomyeloid pathotype, dominated by lymphoid-lineage cells (T, B and plasma cells) in addition to myeloid cells, and a fibroid/pauci-immune pathotype, characterized by few immune cells and prevalent stromal cells. To further dissect synovial cell types, we applied an in silico deconvolution analysis (MCP-counter^10^; Fig. 1b), showing significantly higher CD8 T cells in responders to rituximab and higher macrophage-monocytes and myeloid dendritic cells (mDCs) in responders to tocilizumab (Fig. 1c). Moreover, when we stratified patients according to MCP-counter scores, patients poor in B cells showed significantly higher response rates to tocilizumab (Fig. 1d), consistent with the primary results of the trial^9^, while no difference was found in patients rich in B cells. In contrast, macrophage- and mDC-rich individuals showed higher response to tocilizumab (Fig. 1e). Combined scores (Fig. 1f) demonstrated that patients poor in B cells but rich in macrophages/mDCs had a significantly higher response to tocilizumab (77% responders to tocilizumab versus 14% responders to rituximab; *P* = 0.017, OR = 16.48, 95%CI 1.29–1,000.5). Furthermore, by analysis of disease activity over time, we found a statistically significant interaction effect between treatments and time in patients who were B cell poor (*P* = 0.003), T cell poor (*P* = 0.022) (Fig. 1g), mDC rich (*P* = 0.029) (Fig. 1h) and B cell poor/macrophage/mDC rich (*P* = 0.006) (Fig. 1i). There were significantly lower disease activity scores (clinical disease activity index (CDAI)) at weeks 6, 12 and 16 in patients treated with tocilizumab who were B cell poor and macrophage/mDC rich (Fig. 1i) versus those treated with rituximab. Overall, these results point to myeloid cell infiltration in synovia as one of the key factors explaining the enhanced response to tocilizumab in patients with B cell-poor synovitis.

### Unsupervised clustering defines treatment response diversity

**Extended Data Fig. 2:**
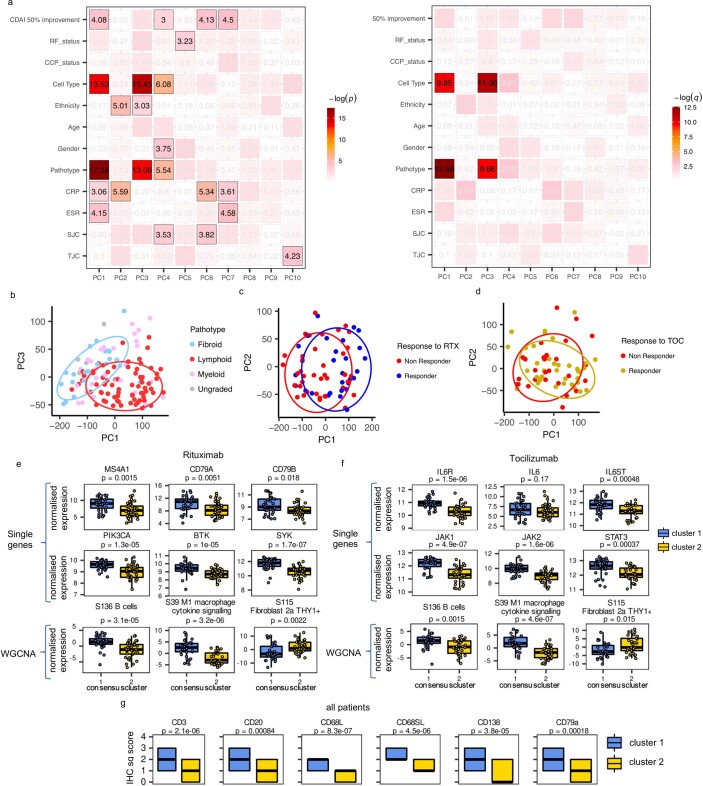
Unsupervised Principal Component Analysis shows association primarily with cell types present and consequently also pathotype. **a**, Clinical features and their degree of association with Principal Components (PC) 1–10 with coloring indicating the –log(p) (left) and FDR corrected –log(q) value (right). RF, Rheumatoid Factor; CCP, anti-Cyclic Citrullinated Protein; CRP, C-Reactive Protein; ESR, Erythrocyte Sedimentation Rate; SJC, Swollen Joint Counts; TJC, Tender Joint Count. **b**, PC 1 and 3 gene expression variance with coloring by (b) pathotypes showing fibroid (blue), lymphoid (red), myeloid (pink) and ungraded (grey) patients. Ellipses indicate 80% confidence interval. **c and d**, PC1 and 2 colored by response to treatment. Patients allocated to treatment group rituximab are displayed in c and to tocilizumab in d, with non response colored in red, response to RTX in blue and response to TOC in gold. Ellipses shown for all PCs represent the 80% confidence interval. **e**, Differential expression of genes important for B-cells (MS4A1, CD79A, CD79B, PIK3CA, BTK and SYK) and Weighted Gene Correlation Network Analysis (WGCNA) cell modules (B-cells, M1 macrophage cytokine signalling, Fibroblast 2a THY1+) in Rituximab treated patients (n = 68), according to the consensus clusters shown in (Fig. 2a). Boxplots show median with upper and lower hinges and whiskers extending to highest and lowest point, but at most 1.5x the interquartile range. p-values stated for Kruskal-Wallis test. **f**, IL-6 related genes (IL6R, IL6, IL6ST, JAK1, JAK2 and STAT3) and WGCNA cell modules expression in tocilizumab (Fig. 2b) treated patients (n = 65) based on consensus clusters. Boxplots as above. **g**, Boxplots showing median with upper and lower hinges for semiquantitative histological scores of CD3, CD20, CD68L, CD68SL, CD138 and CD79a for all patients (n = 133) split into consensuscluster 1 and consensuscluster 2. Kruskal-Wallis test p-values are shown.

Next, we used unsupervised analyses to explore the relationship of multiple genes/pathways with response to treatment. First, we applied principal component analysis (PCA) to identify underlying subgroup structures. PC1 and PC3 correlated with inflammatory cell infiltration in synovial biopsies, while they also associated with histological pathotypes primarily separating the lympho-myeloid and fibroid pathotypes (Extended Data Fig. 2a,b).

**Fig. 2:**
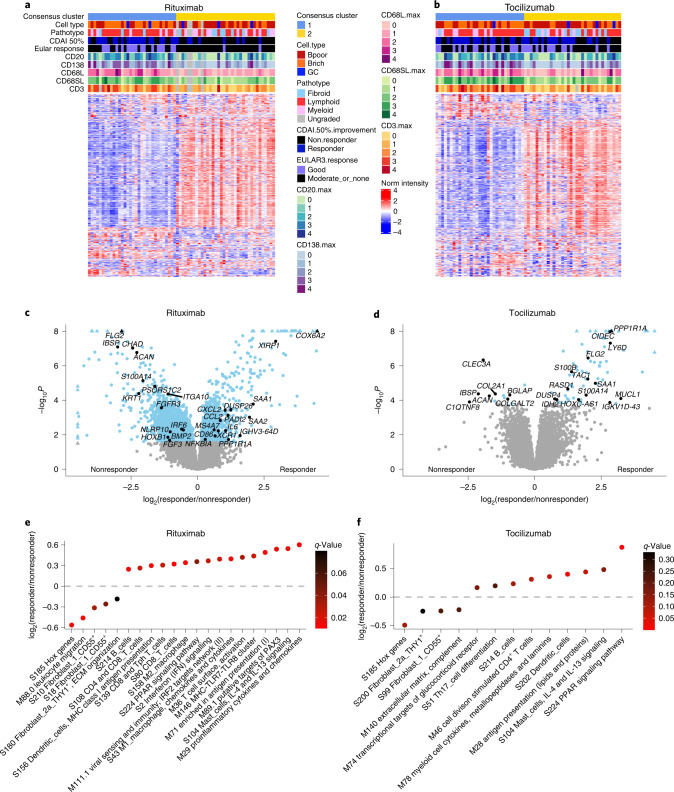
Molecular signatures of response and nonresponse to rituximab and tocilizumab. **a**,**b**, Monte Carlo reference-based consensus clustering of the 22,256 most variable genes identified a high-inflammatory-consensus cluster 1 (blue) and low-inflammatory cluster 2 (yellow). Heatmaps were produced for patients treated with rituximab (*n* = 68, **a**) and tocilizumab (*n* = 65, **b**) using Pearson’s distance metric and the complete linkage method using the ComplexHeatmap package in R. Upper tracks show consensus cluster, cell type (B cell rich/poor), the overall pathotype, CDAI 50% response, EULAR response and histological scores for CD20, CD138, CD68L, CD68SL and CD3. **c**,**d**, Volcano plots of DEGs using DESeq2 comparing CDAI 50% responders versus nonresponders to rituximab (**c**) and tocilizumab (**d**). Comparison between groups using Wald's test and correcting for multiple testing, Storey’s *q*-value (*q* < 0.05 significant, shown in blue). Positive and negative values represent upregulation and downregulation, respectively, in responders and nonresponders. **e**,**f**, Modular analysis applying QuSAGE to responders versus nonresponders to rituximab (**e**) and tocilizumab (**f**); log_2_ fold changes of responders (positive values) and nonresponders (negative values) are plotted for blood microarray-based modules^12^, with WGCNA modules summarized in one plot and dots color coded for their *q*-value.

**Extended Data Fig. 3:**
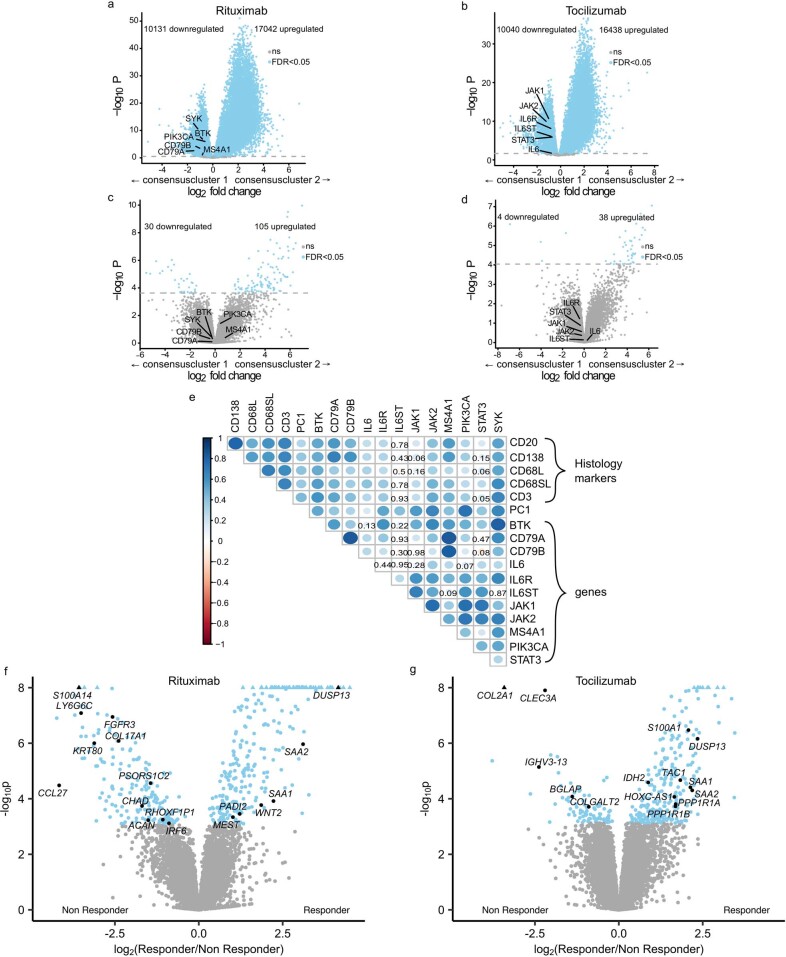
Influence of immune cells on consensusclusters. **a-d**, Volcano plots showing differential gene expression analysis using DESeq2 comparing consensuscluster 1 and 2 of patients treated with rituximab (left) or tocilizumab (right). While **a** and **b** were analyzed without covariates, **c** and **d** were adjusted for principal component (PC1). Comparison between groups were tested for significance using Wald test and multiple testing was corrected for with Storey’s q value (q < 0.05 = significant, shown in blue). Positive log2fold changes represent upregulation in consensuscluster 2, negative log2fold changes represents upregulation in consensuscluster 1. **e**, Correlation plot highlighting relation between PC1, histology markers and genes involved in the mode of action of RTX and TOC. Positive correlation is shown in blue while red would indicate negative correlation. For all correlations without significance, the p-value is shown. **f,g**, Volcano plots of DEGs using DESeq2 comparing CDAI50% responders versus non responders to rituximab (f) and tocilizumab (g) after adjustment for principal component 1. Comparison between groups using Wald test and correcting for multiple testing Storey’s q value (q < 0.05 = significant, shown in blue). Positive values represent upregulation in responders and negative values downregulation compared to non-responders.

Unsupervised Monte Carlo consensus clustering (M3C)^11^ showed 71% of rituximab responders (*n* = 24) in cluster 1 compared with only 29% (*n* = 10) in cluster 2 (*P* = 0.0004; Fig. 2a). Genes relevant for B cell biology were significantly higher in cluster 1 in patients treated with rituximab (Extended Data Figs. 2e and 3a). Cluster 1 was also linked with significant upregulation of the B cell gene module S136 from weighted gene correlation network analysis (WGCNA)^6^, together with upregulation of the proinflammatory M1 macrophage module S39 and downregulation of the fibroblast module S115 (Extended Data Fig. 2e).

Clustering of patients treated with tocilizumab was less distinctive, with 46% of responders (*n* = 21) in cluster 1 and 54% in cluster 2 (*n* = 25) (Fig. 2b). However, cluster 1 was significantly associated with IL-6 pathway genes (Extended Data Figs. 2f and 3b), together with upregulation of B cell and M1 macrophage modules and downregulation of fibroblast modules. In keeping with the increase in immune cell-related modules in cluster 1 for both treatments, semiquantitative IHC scores for synovial immune cells were significantly higher in cluster 1 (Extended Data Fig. 2g), indicating that immune cell infiltration is linked to gene expression in cluster 1, as inferred by the loss of significance when adjusting differentially expressed gene (DEG) analysis between consensus clusters 1 and 2 for immune cell content using PC1 as a covariate (Extended Data Fig. 3c,d). The strong correlation of PC1 with histology markers and immune cell-related genes (Extended Data Fig. 3e) is probably linked to this effect.

### Molecular signatures of treatment response

Next, we performed DEG analysis to identify genes associated with treatment response on all patients who at any point in the trial had received rituximab or tocilizumab (as described in Methods and Supplementary Fig. 1). A total of 6,625 genes were significantly different (false discovery rate (FDR) < 0.05) in rituximab responders compared with nonresponders (Fig. 2c and Supplementary Data 1), and 85 for tocilizumab (Fig. 2d and Supplementary Data 1). Genes upregulated in the synovial tissue of rituximab responders included members of the immunoglobulin (Ig) superfamily and leukocyte-related genes. Nonresponse to rituximab, on the other hand, was associated with complement genes, bone morphogenic proteins, fibroblast-related genes and several Hox genes. Interestingly, lymphocyte and Ig genes were also upregulated in the synovial tissue of tocilizumab responders. Both nonresponder groups showed upregulation of extracellular matrix genes, including integrin-binding sialoprotein, aggrecan and collagen, and genes linked to tissue remodeling, cell infiltration and cell–cell interaction. Following adjustment for immune cell infiltration by PC1, DEGs for rituximab remained significant and, in the case of tocilizumab, the number of identified DEGs increased (Extended Data Fig. 3f for rituximab and Extended Data Fig. 3g for tocilizumab; Supplementary Data1), suggesting that DEG analysis provides an additional dimension to the inflammatory cell infiltrate alone that differentiates responders from nonresponders. Of note, inclusion of covariates such as age, gender and ethnicity was not associated with major differences in the statistical significance of DEGs (Supplementary Data 1).

To investigate the functional role of the above genes, we applied quantitative set analysis for gene expression (QuSAGE) modular analysis^12^ using blood- and synovium-specific WGCNA modules (Fig. 2e,f)^6,13^. Antigen presentation, T and B cell-related modules and interferon signaling were significantly increased in rituximab responders, while Hox gene and fibroblast modules were increased in rituximab nonresponders (Fig. 2e).

Myeloid cell cytokine, peroxisome proliferator-activated receptor (PPAR) and metabolic pathways were upregulated in tocilizumab responders (Fig. 2f). Although none of the modules was significantly modulated in nonresponders to tocilizumab, fibroblast modules were also detected in nonresponders to tocilizumab, suggesting the possible existence of a shared treatment-resistant signature.

### Refractory disease is linked to a stromal/fibroblast signature

**Fig. 3:**
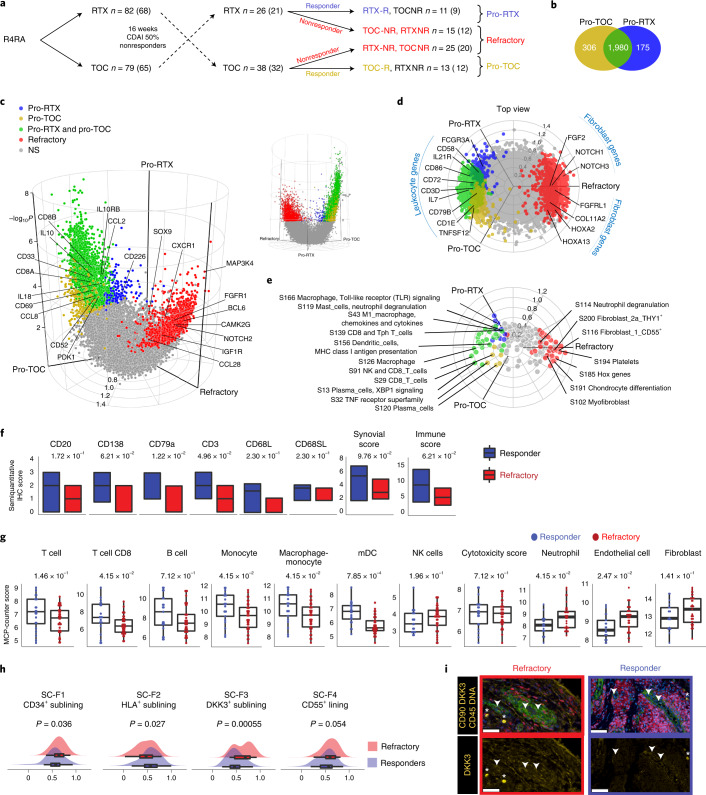
Identification of multidrug nonresponse (refractory) signature. **a**, Patient classification according to treatment switch (complete scheme shown in Supplementary Fig. 1): patients responding to rituximab (RTX) following tocilizumab (TOC) failure (pro-rituximab, blue), patients responding to tocilizumab following rituximab failure (pro-tocilizumab, yellow) and patients in whom both drugs failed sequentially (refractory, red). Numbers in brackets denote patients with available RNA-seq. **b**, Venn diagram showing the overlap of DEGs between patients classified as in **a**. **c**,**d**, Three-way DEG analysis on baseline synovial biopsies of patients classified as in **a**, with side (**c**) and top view (**d**). Significant differences in pro-rituximab (blue), pro-tocilizumab (yellow) and refractory (red) patients and significant genes overlapping in pro-rituximab and pro-tocilizumab patients (green) are color coded. Significance was internally estimated by the volcano3D package combining significance (*q* < 0.05) from both LRT and pairwise Wald test via DESeq2. **e**, Three-way QuSAGE radial plot showing differential WGCNA module expression in patients classified as above. **f**, Histological semiquantitative scores for immune cells in refractory patients (*n* = 40) and responders to one of any two medications (*n* = 24). Boxplots showing median and first and third quartiles. Two-way Mann–Whitney test, exact *P* values FDR adjusted for multiple comparisons. **g**, Deconvolution of immune cells using MCP-counter in patients classified as refractory or responders as in **a**. Boxplots showing median and first and third quartiles, dot-plots showing individual patients. Two-way Mann–Whitney test, exact *P* values FDR adjusted for multiple comparisons. **h**, Fibroblast single-cell subset enrichment scores in refractory patients (*n* = 32) or responders to either rituximab or tocilizumab (*n* = 21), as in **a**. Boxplots showing median and first and third quartiles, whiskers extending to the highest and lowest values. Exact *P* values are shown, two-sided Mann–Whitney test. **i**, Multiplex immunofluorescence in refractory and responder patients; nuclear staining (blue), CD45 (red), CD90 (green), DKK3 (yellow) (all top) and DKK3 single staining (yellow, bottom). *, DKK3^+^CD45^+^ lymphocytes; arrowheads, DKK3^+^CD90^+^ fibroblasts. A larger overview and individual stainings are provided in Extended Data Fig. 4. Representative images out of a total of three refractory and three responders. Scale bars, 50 μm. NS, not significant.

To further explore the hypothesis of a common refractory signature following treatment switch at 16 weeks (Supplementary Fig. 1), we compared patients in whom both rituximab and tocilizumab failed to induce response (multidrug resistant/refractory, *n* = 40 for histology, *n* = 32 for RNA-seq) with (1) patients who responded exclusively to rituximab after tocilizumab failure (pro-rituximab, *n* = 11 for histology and *n* = 9 for RNA-seq) and (2) patients who responded exclusively to tocilizumab after rituximab failure (pro-tocilizumab, *n* = 13 for histology and *n* = 12 for RNA-seq) (Fig. 3a). We identified 1,980 genes upregulated in both pro-rituximab and pro-tocilizumab patients, 175 exclusive to the pro-rituximab group and 306 exclusive to the pro-tocilizumab (Fig. 3b and Supplementary Data 2). Among genes upregulated in responders to both medications were lymphoid, myeloid and many cytokine genes (Fig. 3c,d). Chemokines and lymphocyte genes were upregulated in pro-rituximab patients, while lymphocyte and myeloid lineage genes were upregulated in pro-tocilizumab.

Modular analysis showed antigen presentation and dendritic, macrophage and plasma cell infiltration modules upregulated in responders to both biologics (Fig. 3e). Similarly, the CD8 and Tph T cell module was upregulated in each drug response group, with greater change for the rituximab responder group (proximity to pro-rituximab axis), while Toll-like receptor signaling and macrophage chemokine and cytokine signaling were significantly increased in pro-rituximab patients only. Modules for T cells, plasma cells and the TNF receptor superfamily gene were upregulated in pro-tocilizumab patients only (Fig. 3e).

Notably, 1,277 significant genes were unique to multidrug-resistant/refractory patients (Fig. 3c,d and Supplementary Data 2), including fibroblast and extracellular matrix-encoding genes such as fibroblast growth factor (*FGF*), homeobox (*HOX*) and *NOTCH* family genes, together with multiple cell-adhesion-molecule- and collagen-encoding genes (Fig. 3e and Supplementary Data 3).

In line with molecular signatures, baseline histological scores for CD3^+^ T cells and CD79a^+^ B-cells and CD138^+^ plasma cells were significantly lower in refractory patients (Fig. 3f). Additionally, in silico deconvolution showed significantly lower levels of CD8^+^ T cells, monocytes and mDCs and a trend towards increase in endothelial cells, neutrophils and fibroblasts in refractory patients (Fig. 3g).

To further characterize the association of synovial fibroblast genes with multidrug resistance, we complemented MCP-counter deconvolution by examining enrichment in synovium-specific fibroblast gene modules derived from RA synovial single-cell RNA-seq^14^. As shown in Fig. 3h, the signature for HLA-DRA^high^ sublining fibroblasts (SC-F2), a proinflammatory subset associated with leukocyte-rich synovial infiltration in RA, was significantly higher in responders (*P* = 0.027) as opposed to CD34^+^ sublining fibroblasts (SC-F1) and, in particular, to the newly described DKK3^+^ sublining fibroblasts (SC-F3), both increased in refractory patients (*P* = 0.036 and 0.00055, respectively).

**Extended Data Fig. 4:**
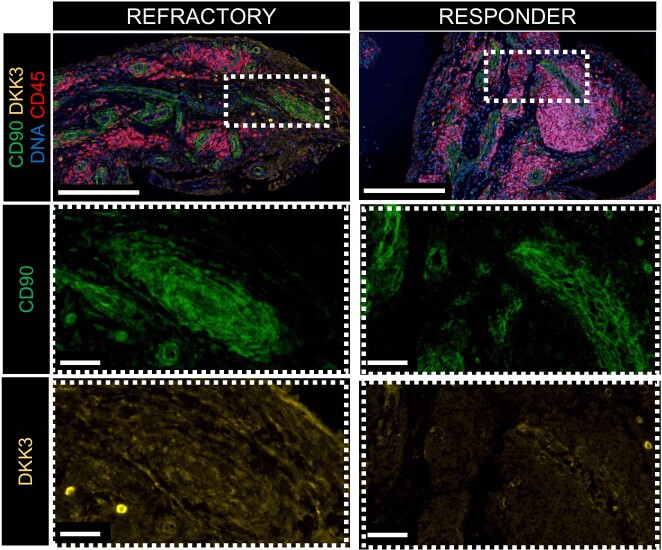
Immunofluorescence of DKK3 + fibroblasts. DKK3 + fibroblasts in refractory (left) and responder (right) patients (representative image out of 3 refractory and 3 responders). Immunofluorescence with DNA in blue, CD45 in red, CD90 in green and DKK3 in yellow. Lines at 0.250 mm in the overview (top panels) and 0.05 mm in the higher magnification (bottom panels).

For orthogonal confirmation of these findings at the protein level, we used multiplex immunofluorescence to detect DKK3^+^ fibroblasts in the synovial lining and sublining of refractory patients (Fig. 3i and Extended Data Fig. 4).

Together, these results show that baseline histological and molecular signatures are associated with response to individual drugs, while nonresponse to multiple biologics is linked to a specific pretreatment signature associated with fibroblasts.

### Digital spatial profiling of refractory RA

**Fig. 4:**
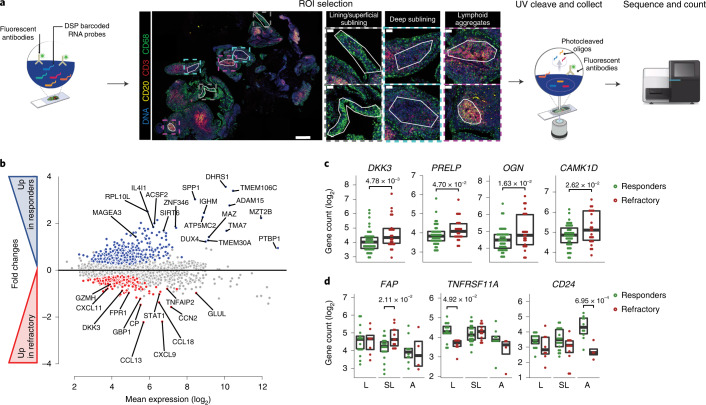
DSP of refractory RA. **a**, Scheme showing the approach to DSP, including selection of ROIs: CD68^+^ lining and superficial sublining, CD20^–^CD3^–^ deep sublining and CD3^+^CD20^+^ lymphoid aggregates. **b**, MA plot showing mean expression (log_2_) on the *x* axis and fold change on the *y* axis comparing responders and refractory patients across all ROIs. Genes significantly upregulated (FDR<0.05) in responders are shown in blue (top), and those upregulated in refractory in red (bottom); in grey, genes with *FDR* > 0.05; *P* values were calculated using a negative binomial linear model applied to count data using DESeq2 (Wald test) and were FDR adjusted *n* = 12 patients, six ROIs per patient. **c**, Example of individual genes differentially expressed in refractory (red) or responders (green). Scatterplots showing individual ROIs, boxplots showing median and first and third quartiles. FDR-adjusted *P* values calculated as in **b** are shown for differentially expressed genes between refractory and responder individuals; *n* = 12 patients (4 responders to rituximab, 4 responders to tocilizimab and 4 refractory). **d**, Examples of individual genes differentially expressed in refractory (red) or responders (green) in different ROIs. Scatterplots showing individual ROIs (*n* = 12 patients, six ROIs per patient), boxplots showing median and first and third quartiles. FDR-adjusted *P* values calculated as in **b** are shown for differentially expressed genes between refractory and responder individuals. L, lining/superficial sublining; SL, deep sublining; A, lymphoid aggregates (as shown in **a**).

Because immune and stromal cells are known to exhibit positional identity relevant to the pathogenesis of RA^15^, we used digital spatial profiling (DSP) to characterize the spatial positioning of cell signatures in association with treatment response/resistance. We employed GeoMx DSP (NanoString), which uses a set of protein lineage markers to define regions of interest (ROIs) that undergo whole-transcriptomic spatial RNA expression (Fig. 4a). First, we compared gene expression in responders and refractory patients across all ROIs: lining/superficial sublining, deep sublining and lymphoid aggregates (Fig. 4b). Consistent with the above bulk RNA-seq modules and protein expression, multiple genes related to the DKK3^+^ fibroblast subset (*PRELP*, *OGN*, *CAM1KD*) were significantly higher in refractory patients (Fig. 4c). When looking at individual ROIs, we found specific genes differentially expressed in responders versus refractory patients in each synovial region. For example, the gene encoding for the fibroblast marker FAP^16^ was significantly upregulated in the deep sublining of refractory patients, the gene for the osteoclast marker RANK (*TNFRSR11A*) was significantly higher in the lining/superficial sublining of responders and *CD24* encoding a lymphocyte marker was significantly higher in the lymphoid aggregates of responders (Fig. 4d).

### Pre- and post-treatment histopathological and molecular analyses

**Fig. 5:**
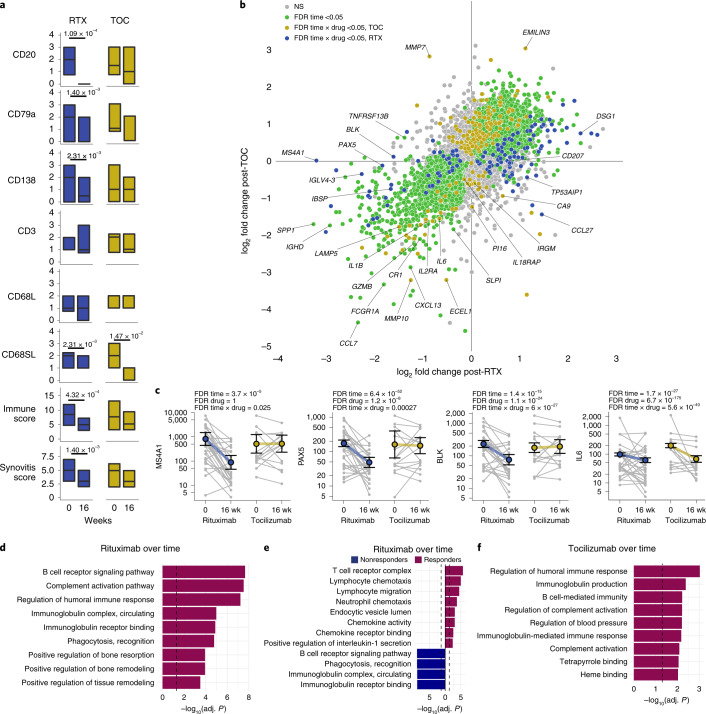
Histological and molecular analysis of paired pre- and post-treatment synovial biopsies. **a**, Semiquantitative histological scores of synovial immune cells at baseline and 16 weeks in patients treated with rituximab and tocilizumab. Boxplots showing median and first and third quartiles. *P* values shown when <0.05, two-sided Wilcoxon signed-rank test (paired) comparing baseline and 16 weeks, adjusted for multiple testing by FDR; *n* = 65 patients with matched baseline and 16-week samples (41 randomized to rituximab, 24 to tocilizumab). **b**, Scatter plots comparing longitudinal gene expression changes between drugs over 16 weeks of treatment in 88 paired biopsies from 44 patients following treatment with rituximab (*n* = 29) or tocilizumab (*n* = 15). log_2_ fold change in expression following rituximab or tocilizumab is represented on the *x* and *y* axis, respectively. Genes equally affected by each drug lie along the line of identity. Fold change and statistical analysis of longitudinal differential gene expression were calculated by negative binomial general linear mixed-effects model. Genes in green show significant (FDR < 0.05) overall change in expression over time; those in blue/yellow show significantly differential change in expression over time between the two drugs based on significant (FDR < 0.05) interaction term *time* *×* *medication* (Methods). Genes with greater absolute fold change following rituximab or tocilizumab are shown in blue and yellow, respectively. **c**, Scatter plots for selected genes with colored points showing regression line of fitted mixed-effects model, with error bars showing 95% CIs (fixed effects). Gray points and lines show raw paired count data, with numbers as per the analysis above. **d**–**f**, Pathway analysis using a two-sided hypergeometric test to enrich downregulated genes between baseline and 16 weeks in patients treated with rituximab (**d**), responders and nonresponders to rituximab (**e**) and responders to tocilizumab (**f**). Dashed line indicates adjusted *P* = 0.05 (Bonferroni adjustment).

**Extended Data Fig. 5:**
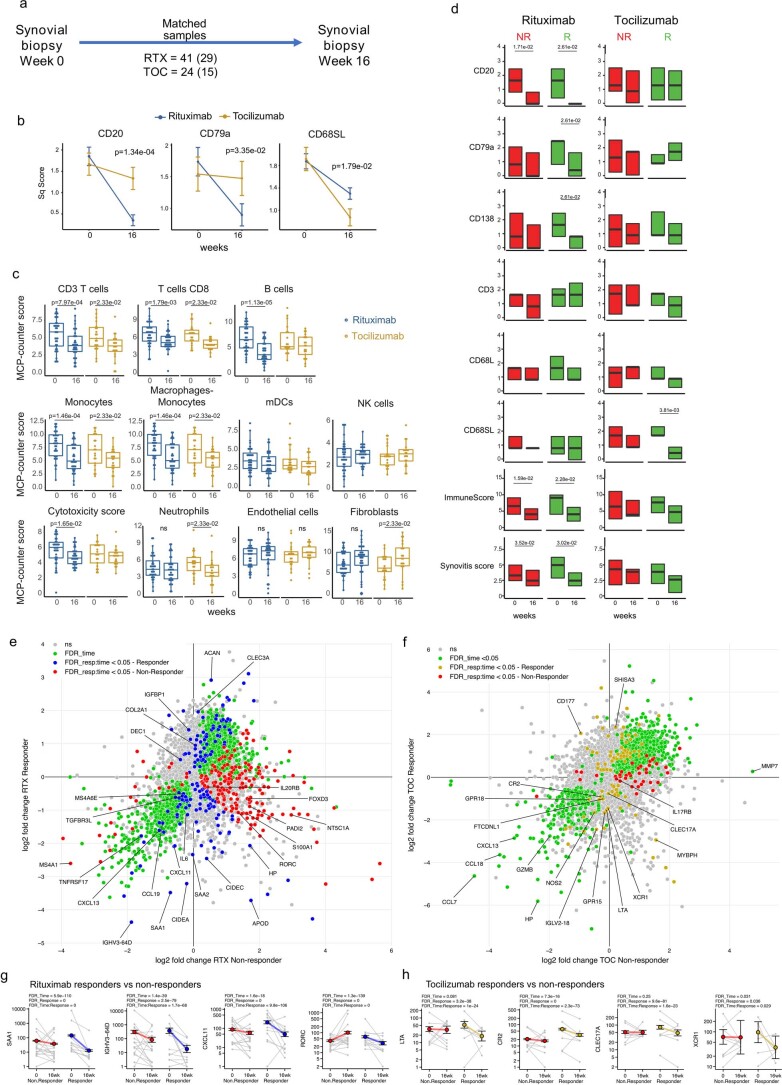
Longitudinal analysis of paired pre- and post-treatment synovial biopsies. **a**, Schema showing an overview of longitudinal analysis of matched pre and post-treatment synovial biopsies, with number of samples for each medication (in brackets samples with available RNA-Seq). **b**, Semi-quantitative scores at baseline and 16 weeks in patients stratified according to treatment with rituximab (n = 41) or tocilizumab (n = 24). Mean ± SEM. Exact p values from two-sided analysis of covariance testing the difference in the changes from baseline between treatments, with treatment as factor and baseline score as covariate. **c**, MCP-counter scores in baseline and 16 weeks samples. Scatterplots showing individual samples and boxplots showing median and first and third quartiles, whiskers extending to the highest and lowest values no further than 1.5*interquartile range. Two-sided Wilcoxon signed-rank test (paired), comparing baseline and 16 weeks, adjusted for multiple testing by false discovery rate. n = 29 for rituximab and n = 15 for tocilizumab. **d**, Semi-quantitative scores of synovial immune cells at baseline and 16 weeks in patients treated with rituximab (n = 41) and tocilizumab (n = 24), stratified by CDAI50% response (NR = non responders, R = responders). Boxplots showing median and first and third quartiles. p values shown when <0.05, two sided Wilcoxon signed-rank test (paired) comparing baseline and 16 weeks, adjusted for multiple testing by false discovery rate. **e,f**, Longitudinal negative binomial mixed effects model on Rituximab (n = 29) and (f) Tocilizumab (n = 15) treated patients showing differential gene expression between responders and non-responders categorised by CDAI 50% response. Blue genes show greater absolute gene expression change in rituximab responders, yellow genes show greater absolute gene expression change in tocilizumab responders, while red genes showed greater absolute gene expression change in non-responders. **g,h** Scatter plots of representative genes with coloured points showing regression line of fitted negative binomial mixed effects model with error bars showing 95% confidence intervals (fixed effects) from analyses in e & f respectively. Grey points and lines show raw paired count data. n = 29 for rituximab and n = 15 for tocilizumab.

To explore the longitudinal effects of each drug on synovial immune cell infiltration and gene expression, we compared paired synovial samples at baseline and 16 weeks (rituximab, *n* = 41 for histology and *n* = 29 for RNA-seq; tocilizumab, *n* = 24 and *n* = 15, respectively) (Extended Data Fig. 5a and Extended Data Table 1). First, by histology, we showed a significant reduction in synovial CD20^+^ total B cells, CD79a^+^ B cells and CD138^+^ plasma cells in patients treated with rituximab, in line with the rituximab mechanism of action targeting CD20^+^ B cells (Fig. 5a). Conversely, patients treated with tocilizumab showed a significant reduction in CD68^+^ sublining macrophages but not B-cells (Fig. 5a). Analysis of covariance showed a significantly higher reduction of CD20^+^ and CD79a^+^ B cells in patients treated with rituximab, and a significantly higher reduction in CD68^+^ sublining macrophages in those treated with tocilizumab (Extended Data Fig. 5b).

Similar results were obtained when comparing MCP-counter immune cell signatures. Namely, patients treated with rituximab showed a significant reduction in B cells, T cells and monocytes/macrophages while those treated with tocilizumab showed a significant reduction in monocytes/macrophages and T cells, but also in neutrophils and mDCs and, interestingly, an increase in fibroblast signature (Extended Data Fig. 5c). This suggests that both biologics have an effect on immune cells but that tocilizumab can potentially also affect stromal cells.

To further dissect these longitudinal molecular signatures, we developed an R package to fit negative binomial mixed-effects models at the individual gene level (general linear mixed-effects model (glmmSeq)), because mainstream RNA-seq analysis tools are unable to fit mixed-effects linear models (Methods).

Using glmmSeq to compare gene expression over time in paired synovial biopsies, 7,316 genes were significantly up- or downregulated by both drugs while 345 were differentially affected by either drug based on significance (FDR < 0.05) of the interaction term *time* *×* *medication* (Fig. 5b and Supplementary Data 4a). Of note, *MS4A1* (encoding CD20), *PAX5* and *BLK* were significantly downregulated in response to rituximab, consistent with B cell depletion mechanism and histology results (Fig. 5c), while tocilizumab induced a reduction in IL-6-related transcripts, also consistent with the IL6 acting mechanism of tocilizumab, not the CD20 mechanism of rituximab.

When patients were stratified according to response, a significant reduction in CD138 and CD79a plasmablasts/plasma cells was observed only in rituximab responders, while a significant reduction in CD68SL macrophages was observed only in responders to tocilizumab (Extended Data Fig. 5d), indicating that reduction in B and synovial plasma cells and macrophages is associated with response to rituximab and tocilizumab, respectively.

The mixed-effects model allowed us to further examine various changes in gene expression following therapy between responders and nonresponders to each drug (Extended Data Fig. 5e–h). Rituximab had a general effect on 1,796 genes, with 349 showing significant (FDR < 0.05) differential expression change over time between responders and nonresponders (Extended Data Fig. 5e and Supplementary Data 4b). Rituximab responders showed a greater decrease in *SAA1* and *SAA2* (serum amyloid proteins 1 and 2), as well as greater decreases in Ig chain genes *IGHV3-64D* and *IGKV1-13*, suggesting that a drop in antibody-secreting B cells is associated with response to rituximab (Extended Data Fig. 5g). Chemokine-encoding *CXCL11*, the citrullination enzyme encoding *PADI2* (peptidyl-arginine-deiminase2) and the key Th17 and mucosal-associated invariant T (MAIT) cell transcriptional regulator RORgamma (*RORC*) gene were also modulated in rituximab responders (Extended Data Fig. 5g).

Tocilizumab treatment resulted in modulation of 1,609 genes, with an additional 136 showing differential change in gene expression between responders and nonresponders (Extended Data Fig. 5f and Supplementary Data 4c). Reduction in pro-lymphoid follicle development genes encoding for lymphotoxin-A (*LTA*), complement receptor-2 (*CR2*), lymphoid-tissue-resident dendritic cell marker *XCR1*^17^ and prolactin (*CLEC17A*), expressed on proliferating germinal center B cells^18^, augured response to tocilizumab (Extended Data Fig. 5h).

To further investigate pathway modulation induced by treatment, genes identified in the longitudinal mixed-effects model analysis were analyzed for Gene Ontology (GO)/pathway enrichment. Rituximab treatment induced significant downregulation of B cell receptor pathways, as well as bone resorption and remodeling pathways (Fig. 5d). When stratifying patients according to response, responders to rituximab showed significant downregulation of T cell receptor complex, lymphocyte chemotaxis and migration, chemokines and IL-1-related pathways (Fig. 5e), suggesting that rituximab response is linked to additional immunomodulation in addition to reduction in B cell-related pathways. Responders to tocilizumab showed significant decrease in humoral immune response, Ig, B cell and complement activation (Fig. 5f), in line with the known effect of IL-6 on B cell growth/differentiation.

In summary, longitudinal analyses of matched pre- and post-treatment biopsies indicate that specific biological changes are associated with response to individual treatments.

### Machine learning models predict drug response and multidrug resistance

**Fig. 6:**
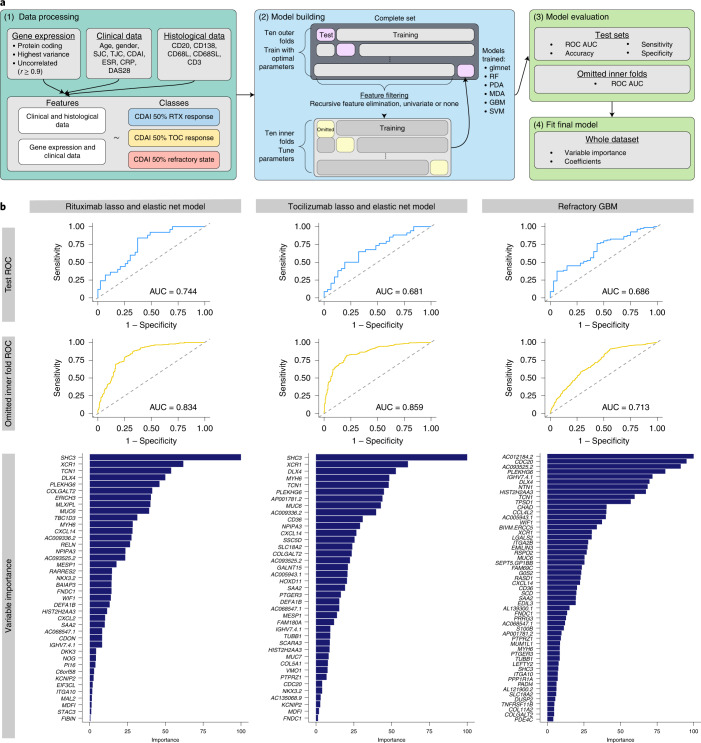
Predictive models using nested ten-by-ten-fold cross-validation for response to rituximab and tocilizumab. **a**, Machine learning pipeline utilized to predict CDAI 50% response to rituximab and/or tocilizumab using gene expression, clinical data and histological data as features (*n* = 133). Data processing (1) involved selection of protein-coding genes with the highest variance and removal of highly correlated genes. Data were split into ten inner and ten outer folds for building machine learning models (2). In models built using gene expression, RFE or univariate filtering was used to select the most important/predictive features for each model. Each model was evaluated on both the test set and the set omitted during cross-validation (3). Average tuned parameters from the outer folds were used to fit to the whole dataset to determine the importance of features selected for each model (4). **b**, Grid of plots showing optimal predictive models for different treatments (left, glmnet rituximab response prediction; middle, glmnet tocilizumab response prediction; right, GBM refractory response prediction) using gene expression and baseline clinical parameters as features. From top to bottom, plots show ROC curves for the best model on the test dataset (from outer fold), ROC curves on the omitted dataset (from inner fold) and variable importance when fit to the whole dataset.

To establish the ability of synovial tissue gene expression in prediction of treatment response/resistance, we developed machine learning predictive models with the dataset partitioned for training and testing using ten-by-tenfold nested cross-validation, as detailed in Methods and schematically in Fig. 6a. Supplementary Table 1 shows the performance of models used to predict (1) rituximab response, (2) tocilizumab response and (3) refractory state. Final models (Supplementary Table 2) were trained on the entire dataset to extract variable importance (Fig. 6a, bottom right and Extended Data Table 2).

**Extended Data Fig. 6:**
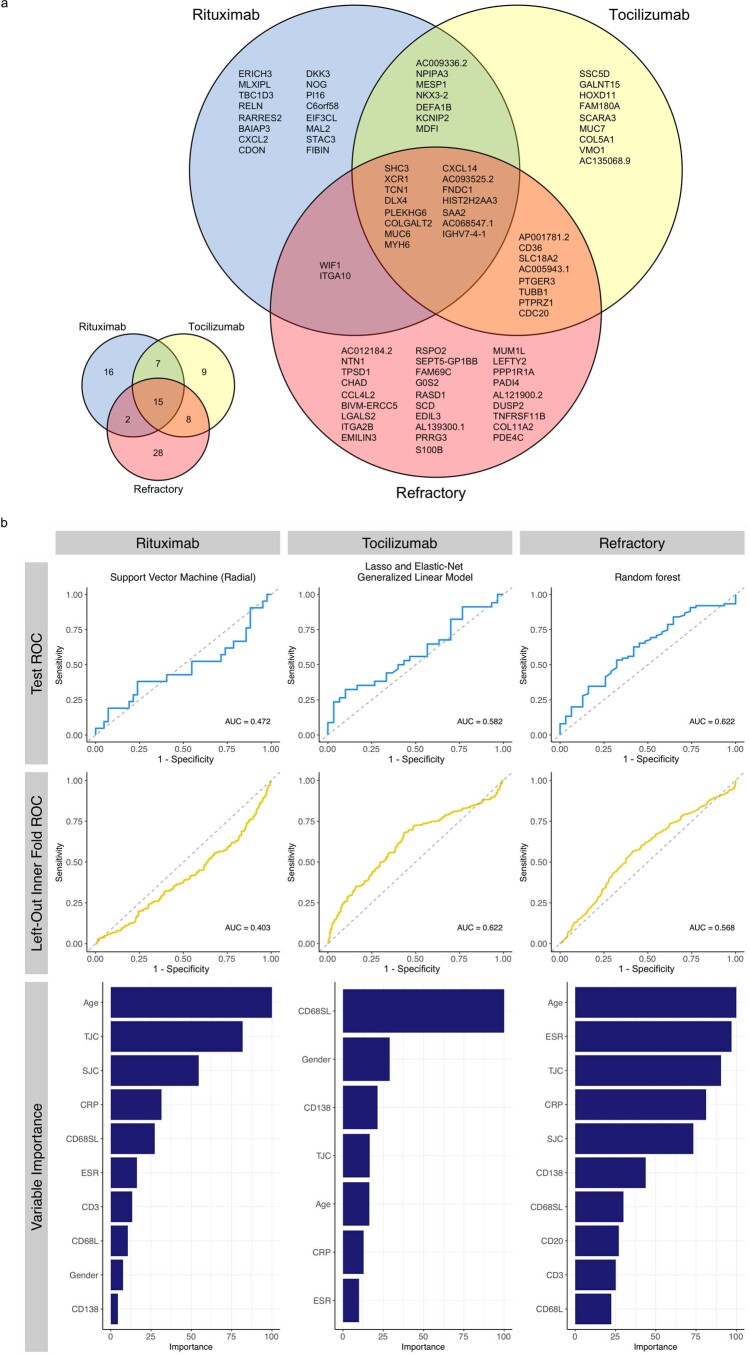
Venn diagram showing overlap in genes between machine learning models and comparison with models built using only clinical and histological variables. **a**, Venn diagram showing the overlap in genes selected as features in optimal predictive models for prediction of rituximab and tocilizumab response at week 16 and refractory state (failure to respond to both rituximab and tocilizumab). **b**, Grid of plots showing the optimal predictive models for different treatment when using clinical and histological variables only. From top to bottom plots show: ROC curves for the best model on the test data (from outer-fold) set; ROC curves on the left-out (from inner-fold) set; and the variable importance when fit to the whole data set.

As shown in Fig. 6b, the optimal predictive models included gene elastic net regression for rituximab and tocilizumab response, with 40 genes (AUC = 0.744) and 39 genes (AUC = 0.681), respectively, and gradient-boosted machine (GBM) for refractory state, with 53 genes (AUC = 0.686) (Extended Data Fig. 6a). Notably, no clinical features were selected by the final models (Extended Data Table 2) and, in comparison with RNA-seq, predictive models built using clinical and histology parameters alone performed quite poorly (Extended Data Fig. 6b). AUC values in the omitted inner cross-validation folds were consistent with AUC results in the true test folds. Multiple genes were shared across models, with only 85 required to build all three prediction models and 32 shared between at least one model (Extended Data Fig. 6a and Extended Data Table 2). Each model selected multiple genes of biological relevance to synovial tissue inflammatory and repair responses, as well as to bone and cartilage biology. Key prediction genes shared between all three models included genes encoding for: *XCR1*, a marker of DC1 migratory DCs;^17^ chemokine *CXCL14*; acute phase reactant *SAA2*; and *IGHV7-4-1*, probably reflecting tissue-resident plasma cells. The refractory state model, which contained the largest number of unique genes, included several linked to the fibroid pathotype such as *TNFRSF11B*, which encodes the osteoclast negative regulator osteoprotegerin and the chondrocyte adhesion mediator *CHAD* (chondroadherin), but also the citrullination enzyme PAD4 encoding gene *PADI4*^19^, consistent with a role of persistent tissue destruction and remodeling in the refractory RA state.

Although the original clinical trial was not powered for these types of analyses, and larger cohorts will be required to further validate the models and improve their predictive power, these results show that predictive models can harness molecular information from synovial biopsies at baseline before treatment and thus are of potential clinical utility for prediction of response to therapy.

## Discussion

Our study provides an in-depth molecular and histological profiling of joint tissue from a biopsy-driven, randomized clinical trial in RA (R4RA)^9^, affording insights into the cellular and molecular pathways underpinning the diverse treatment response to two commonly used targeted biologic therapies directed against B cells (rituximab) and the IL-6 receptor (tocilizumab). Using both conventional histology and in silico deconvolution, we observed that lymphoid cells were associated with response to rituximab while myeloid cells were associated with response to tocilizumab. Although the importance of synovial macrophages as a predictor of response to anti-TNF has been described^20,21^, here we report that within the synovial B cell-poor group, an enhanced response to tocilizumab is associated with the presence of myeloid cells. In regard to rituximab, previous small observational studies reported pretreatment synovial CD79a^+^ B cells^22^ and synovial molecular signatures^23^ as potential response predictors.

In this larger, randomized clinical trial cohort we confirmed some of these findings and, combining histological assessment with advanced molecular analyses, we identified genes and pathways linked to the cognate drug targets in association with response. For rituximab these were B cell genes, Igs, chemokines and leukocyte genes. Response to tocilizumab was associated with IL-6 pathway genes, but also with lymphocyte and immunoglobulin genes, which is not surprising since IL-6 is a well-known B cell growth factor^24^.

Modular analyses demonstrated how genes increased in rituximab responders were functionally related to antigen presentation and lymphocyte activation, together with interferon signaling genes, in keeping with previous reports linking increased type I interferon with response to rituximab^25,26^. In tocilizumab responders, in line with the prominent role of myeloid cells identified by histopathology, the myeloid cell cytokine module was upregulated together with PPAR signaling and metabolic pathways.

In contrast, nonresponse to both drugs was defined by >1,000 genes and several shared pathways, including Hox, FGFs and ECM genes/modules. Critically, this shared nonresponse signature is linked to the fibroid pauci-immune pathotype characterized by scanty immune cell infiltrate with prevalence of stromal cells^6,7^, which we also reported as being associated with poor response to synthetic disease-modifying antirheumatic drugs (DMARDs)^27^ and TNF inhibitors^28^. This supports the concept that the fibroid pauci-immune phenotype represents a refractory endotype, since in R4RA double nonresponder patients conventional synthetic DMARDs (csDMARDs) and TNF inhibitors had already failed, as per trial entry criteria, thus displaying resistance to three biologic therapies targeting distinct immunological pathways (TNF, CD20^+^ B cells and IL6R) and meeting the definition of multidrug resistant/refractory RA^2^.

Because recent studies using single-cell RNA-seq (scRNA-seq) on RA synovium identified specific fibroblast subsets with critical roles in RA pathogenesis^13,14,16,29^, we applied modular analysis based on scRNA-seq fibroblast subsets^14^ and orthogonal validation by multiplex immunofluorescence, and identified an association between DKK3^+^ fibroblasts and refractoriness. *DKK3* encodes Dickkopf3, a negative regulator of beta-catenin that has been shown to promote aggressive behaviour in cancer-associated fibroblasts^30^, although the exact function of DKK3^+^ fibroblasts in RA remains to be established^31^. Furthermore, DSP revealed specific upregulation of genes in the sublining of refractory patients encoding for the fibroblast marker FAP, which has been linked with RA pathogenesis^16^, while other markers consistently modulated across all regions included *CCL13* encoding for the monocyte-attracting chemokine MCP-4, which has been shown to activate synovial fibroblasts^32^. Hence, stromal cells—and, in particular DKK3^+^ fibroblast genes—may be a new drug target that helps overcome the complex problem of refractoriness in RA.

Genes linked with response included increased expression in the synovial aggregates encoding for CD24, a B cell marker associated with response to biologic treatment^33^. Also consistent with the capacity of biologic therapies to halt structural damage progression^34^, we identified increased expression of RANK transcripts (*TNFRSF11A*) in the lining/superficial sublining of responders, in line with the reported presence of osteoclast precursors in inflamed synovia^35^. Overall, these results suggest that the spatial organization of immune infiltrates is highly relevant for determinion of treatment response/resistance, although additional work is needed to dissect the contribution of specific markers in individual synovial regions and their association with therapy response/resistance.

The longitudinal analysis of matched pre-/post-treatment synovial biopsies enabled us to investigate drug effects on synovial pathology and gene expression. Rituximab reduced synovial CD20^+^ B cells in both responders and nonresponders but, notably, a clinically relevant response required broader and deeper impacts on differentiated CD79a^+^ plasmablasts and CD138^+^ plasma cells over and above CD20^+^ B cell depletion. These observations are in agreement with previous observational studies showing changes in plasma cells between responders and nonresponders to rituximab^36^. However, another study showed variable depletion of B cells and plasma cells and an unclear association with treatment response^37^, although the small sample size and biopsy analysis at different time points (4, 8, 12 and 16 weeks) make it difficult to draw conclusions. In our study, because repeated synovial biopsy was performed at 16 weeks following one cycle of rituximab (2× 1-g infusion), it is plausible that a repeated biopsy at the time of the second infusion at 6 months (not available in sufficient numbers to be informative) could have detected a wider/deeper effect on B cell lineages linked to clinical response.

Following tocilizumab therapy, nonresponders were characterized by a failure to reduce sublining macrophages, which is consistent with previous literature indicating macrophages as markers of treatment response^20^.

To assess the relationship of gene expression changes and treatment response, we developed a pipeline for mixed-model analysis of RNA-seq data in repeat biopsies. This revealed patterns of change in gene expression not detectable by standard analytical pipelines, while interaction analysis allowed us to identify genes that were affected by each drug specifically. Biological differences in synovial gene expression following treatment with rituximab or tocilizumab were consistent with the cognate treatment targets: B cell depletion and IL-6 receptor blockade, but also revealed unexpected differences such as differential changes in metalloproteinases. Interaction analysis showed that rituximab responders had a greater decrease in genes encoding for serum amyloid proteins, Ig chains, the citrullination enzyme PADI2 and transcriptional regulator RORgamma. In tocilizumab responders, a greater reduction in pro-lymphoid follicle development genes was observed in keeping with the important role of IL-6 as a B cell growth factor driving in situ ectopic lymphoid structure development within inflamed tissues^38^.

In translational terms, the importance of molecular studies is measured by their ability to enhance disease understanding, but also on their clinical impact^39^. Thus, to determine the predictive value of deep molecular characterization in foretelling treatment response, we applied a number of machine learning methodologies resulting in the selection of models effective at predicting treatment response as tested by nested cross-validation (AUC for rituximab = 0.744, tocilizumab = 0.681, refractoriness = 0.686). The purpose of testing multiple models was to determine whether nonlinear algorithms (least-squares support vector machine (SVM), penalized discriminant analysis (PDA), mixture discriminant analysis (MDA), tree-based and so on) could outperform elastic net linear regression. In practice, for prediction of response to each drug individually, elastic net regression performed best. However, a GBM model was superior in predicting refractoriness, consistent with the notion that biological heterogeneity underlying refractoriness to multiple drugs might require a nonlinear algorithm for optimal prediction. Of relevance to future clinical practice, gene expression models were clearly superior to those built using clinical and histological data alone. Seropositivity, which has been weakly associated with response to rituximab^40^ was not included in the final models.

A known limitation of this study is the relatively modest sample size of the training data for each predictive model. However, the study was powered purely for the primary outcome of the original clinical trial and numbers reflect the difficulty in conducting biopsy-driven randomized trials in RA. With the current sample size and case/biomarker ratio, statistical theory suggests that we are likely to be only part of the way up the learning curve^41^. The AUCs derived in this study would still be relatively low for direct application to clinical use without further validation and improvement. Refining and reducing the number of genes in the models with validation in independent cohorts could improve the prediction models, as could incorporation of information from single-cell studies and enhanced deconvolution methods. Another limitation with regard to interpretation of the longitudinal results is that the second biopsy at 16 weeks was an optional procedure and, thus, at risk of selection bias. Although there were no major baseline differences, patients who underwent repeated biopsy had lower response rates, which is expected because responders would have been less likely to consent to a second biopsy.

In conclusion, this study provides insights from analysis of diseased tissue regarding the mechanisms driving treatment response heterogeneity in RA, and underscores the importance of integrating predictive molecular pathology signatures into clinical algorithms to optimize the usage of existing drugs. The identification of genes and cell types associated with multidrug resistance could aid the development of new drugs for refractory patients in whom current medications targeting classical immune pathways are not effective. We envisage that routine use of synovial biopsies could facilitate a patient-centered approach^5^ to the management of RA, thus moving away from the current trial-and-error drug prescribing towards an emergent era in which selection of the optimal drug is based on synovial biopsy gene signatures.

## Methods

### Patients and intervention

A total of 164 patients aged 18 years or over, fulfilling the 2010 American College of Rheumatology/European Alliance of Associations for Rheumatology (EULAR) classification criteria for RA and who were eligible for treatment with rituximab therapy according to UK NICE guidelines—that is, failing or intolerant to csDMARD therapy and at least one biologic therapy (excluding trial IMPs), were recruited when fulfilling the trial inclusion/exclusion criteria (for the full study protocol and baseline patient characteristics see Humby et al.^9^). Briefly, patients underwent synovial biopsy of a clinically active joint at entry to the trial, performed according to the expertise of a local center as either an ultrasound-guided or arthroscopic procedure^42^ Following synovial biopsy, patients were randomized to receive rituximab as two 1,000-mg intravenous infusions 2 weeks apart or intravenous tocilizumab at a dose of 8 mg kg^−1^ administered at 4-weekly intervals. Patients were followed up every 4 weeks throughout the 48-week trial treatment period, during which RA disease activity measurements and safety data were collected. An optional repeated synovial biopsy of the same joint sampled at baseline was performed at 16 weeks (Supplementary Tables 3 and 4). The study protocol has been published online (http://www.r4ra-nihr.whri.qmul.ac.uk/docs/r4ra_protocol_version_9_30.10.2017_clean.pdf) and was registered on the ISRCTN database (no. ISRCTN97443826) and with EudraCT (no. 2012-002535-28). Patient demographics are reported in the original trial publication^9^. All patients provided written informed consent. Participants did not receive any compensation, except for reimbursement of travel expenses. The study was done in compliance with the Declaration of Helsinki, International Conference on Harmonization Guidelines for Good Clinical Practice and local country regulations. The protocol was approved by the institutional review board of each study center or relevant independent ethics committee (UK Medical Research and Ethics Committe, reference no. 12/WA/0307). The complete list of ethics committees to have approved the protocol is reported below.

**UK ethics committee**

- Wales REC 3 (formerly REC for Wales)

**Local ethics committees at EU sites**

- Comité d’Ethique Hospitalo-Facultaire
- Comissão de Ética para a Investigação Clínica
- Comitato Etico Interaziendale AOU ‘Maggiore della Carità’ di Novara, ASL BI, ASL NO, ASL VCO
- Commissie Medische Ethiek UZ KU Leuven/Onderzoek
- Comité Ético de Investigación Clínica del Hospital Clínic de Barcelona
- Comitato Etico, Fondazione IRCCS Policlinico San Matteo
- Regione Autonoma della Sardegna Azienda Ospedaliero Universitaria di Cagliari Comitato Etico Indipendente

**Complete list of participating sites for data collection:**

- Mile End Hospital and Whipps Cross Hospital, Bart’s Health NHS Trust, London, UK
- Cliniques Universitaires Saint Luc, Louvain, Belgium
- Santa Maria Hospital, Lisbon, Portugal
- Azienda ospedaliera Maggiore della Carità, Novara, Italy
- University Hospital of Wales, Cardiff and Vale University Health Board, Cardiff, UK
- Royal Victoria Infirmary, Newcastle upon Tyne Hospitals NHS Foundation Trust, Newcastle upon Tyne, UK
- Southampton General Hospital, University Hospital Southampton NHS Foundation Trust, Southampton, UK
- Basildon University Hospital, Mid and South Essex NHS Foundation Trust (formerly Basildon and Thurrock University Hospital NHS Foundation Trust), Basildon, UK
- Hospital Clínic de Barcelona, Barcelona, Spain
- Southend University Hospital, Mid and South Essex NHS Foundation Trust (formerly Southend University Hospital NHS Foundation Trust), Southend, UK
- Chapel Allerton Hospital, Leeds Teaching Hospitals NHS Trust, Leeds, UK
- Azienda Ospedaliero Universitaria di Cagliari, Cagliari, Italy
- Homerton University Hospital, Homerton University Hospital NHS Foundation Trust, London, UK
- Nuffield Orthopaedic Hospital, Oxford University Hospitals NHS Foundation Trust, Oxford, UK
- Aintree University Hospital, Aintree University Hospital NHS Foundation Trust, Liverpool, UK
- Manchester Royal Infirmary, Manchester University NHS Foundation Trust, Manchester, UK
- Guy’s Hospital, Guy’s and St Thomas’ NHS Foundation Trust, London, UK
- Fondazione I.R.C.C.S. Policlinico San Matteo, Pavia, Italy
- Universitair Ziekenhuis Leuven, Leuven, Belgium

### Response criteria and treatment switch

The primary endpoint was defined as CDAI ≥ 50% improvement from baseline at 16 weeks^9^. CDAI is calculated by totaling the number of tender joints (0–28), the number of swollen joints (0–28), patient global health assessment on a 0–10 visuoanalogic scale and the care provider global health assessment on a 0–10 visual analog scale.

As shown in Supplementary Fig. 1, CDAI 50% nonresponders at 16 weeks were switched to the alternative biologic agent and their response was assessed at 16 weeks following the switch, as determined by CDAI 50% improvement. Including crossover patients, a total of 108 patients were treated with rituximab and 117 with tocilizumab. Of those treated with rituximab, 43 were defined responders (40%) while 53 responded to tocilizumab (45%). Among all responders, 11 responded to rituximab following tocilizumab failure and were classified as exclusive responders to rituximab (pro-rituximab), while 13 responded to tocilizumab following rituximab failure and were thus classified as pro-tocilizumab. Patients in whom both drugs failed throughout the study were classified as multidrug resistant/refractory (*n* = 40).

### Histological analysis

A minimum of six synovial biopsies were processed in an Excelsior tissue processor before being paraffin-embedded en masse at Queen Mary University of London Core Pathology department. Tissue sections (3–5 µm thickness) were stained with hematoxylin and eosin and IHC markers CD20 (B cells), CD138 (plasma cells), CD21 (follicular dendritic cells) and CD68 (macrophages) in an automated Ventana Autostainer machine. CD79A (B cells) and CD3 (T cells) staining was performed in-house on deparaffinized tissue following antigen retrieval (30 min at 95 °C), followed by peroxidase- and protein-blocking steps. Primary antibodies (CD79A (clone JCB117, Dako), CD3 (clone F7.238, Dako), CD20 (clone L26, Dako), CD68 (clone KP1, Dako) and CD138 (clone MI15, Dako)) were used for 60 min at room temperature. Visualization of antibody binding was achieved by 30-min incubation with Dako EnVisionTM+ before completion by the addition of 3,3′-diaminobenzidine (DAB) + substrate chromogen for 10 s, followed by counterstaining with hematoxylin. Following IHC staining, sections underwent semiquantitative scoring (0–4), by a minimum of two assessors, to determine levels of CD20^+^ and CD79a^+^ B cells, CD3^+^ T cells, CD138^+^ plasma cells and CD68^+^ lining (L) and sublining (SL) macrophages, adapted from a previously described score^43^ and recently validated for CD20^44^. Hematoxylin-and-eosin-stained slides also underwent evaluation to determine the level of synovitis according to the Krenn synovitis score (0–9)^45^. The sum of semiquantitative scores for Krenn synovitis score (0–9), CD20 (0–4), CD3 (0–4), CD138 (0–4) and CD68 (0–4) is reported as the immune score (0–25). Synovial biopsies were classified into synovial histological patterns, also known as pathotypes, according to the following criteria: (1) lymphomyeloid presence of grade 2–3 CD20^+^ aggregates, CD20 ≥2 and/or CD138 ≥2; (2) diffuse-myeloid CD68SL ≥2, CD20 ≤1 and/or CD3 ≥1 and CD138 ≤2; and (3) pauci-immune-fibroid CD68SL <2 and CD3, CD20 and CD138 <1.

### RNA-seq and molecular classification/analysis

A minimum of six synovial samples per patient were immediately immersed in RNA-Later and RNA was extracted from tissue using one of two protocols: phenol/chloroform isolation and Zymo Direct-zol RNA MicroPrep–Total RNA/miRNA Extraction kit. In both methods, tissue was lysed in Trizol solution using a LabGen125 homogenizer. Briefly, for the phenol/chloroform extraction method, 1–10 mg of tissue was lysed and then sheared using a 21 G needle. The tissue lysate was then mixed vigorously with chloroform before centrifugation. The aqueous phase was removed and mixed with ice-cold isopropanol for 30 min. Following further centrifugation, the RNA pellet was washed in 70% ethanol before air-drying and resuspension in RNAse-free water. Samples extracted using Zymo Direct-zol Miniprep kits were processed as per the manufacturer’s instructions. Briefly, 1–10 mg of tissue lysate was run through the Zymo-Spin IC column. Columns were then washed using the appropriate kit wash buffers before RNA was eluted and resuspended in RNAse-free water. Quality control was carried out by quantifying samples via spectrophotometer readings on a Nanodrop ND2000C. RNA integrity was measured using Pico-chip technology on an Agilent 2100 Bioanalyzer to determine RNA integrity number. A total of 214 synovial tissue samples were available for RNA extraction and were subsequently sent for RNA-seq to Genewiz. RNA-seq libraries were prepared using the NEBNext Ultra RNA Library Prep kit for Illumina, following the manufacturer’s (NEB) instructions. Briefly, messenger RNAs were initially enriched with Oligo d(T) beads followed by limited PCR cycles. The sequencing library was validated on an Agilent TapeStation (Agilent Technologies) and quantified using a Qubit 2.0 Fluorometer (Invitrogen), as well as by quantitative PCR (KAPA Biosystems). The sequencing libraries were clustered on Illumina flowcells. Sequencing was performed on an Illumina HiSeq instrument according to the manufacturer’s instruction. Samples were sequenced using a 2 × 150-base-pair (BP) paired-end configuration.

### RNA-seq data processing

A total of 214 paired-end RNA-seq samples from 50 million reads of 150-bp length were trimmed to remove the Illumina adapters using bbduk from the BBMap package v.37.93, with default parameters. Transcripts were then quantified using Salmon^46^ v.0.13.1 and an index generated from the Gencode release 29 transcriptome following the standard operating procedure. Tximport v.1.13.10 was used to aggregate transcript-level expression data to genes, then counts were subjected to variance-stabilizing transformation (VST) using the DESeq2 v.1.25.9 package^47^. Following RNA-seq quality control, 36 samples were excluded due to poor mapping or RNA quality. Using unsupervised PCA and plotting the first five eigenvectors in pairs, one outlier was identified and removed from further analysis. Thus RNA-seq data from 133 patients were available for subsequent analysis at baseline, and from 44 patients for the follow-up time point. Baseline characteristics of patients with available RNA-seq are shown in Supplementary Table 5. The first six PCs did not associate with demographics, treatment and its associated response or clinical disease features such as disease activity or anticyclic citrullinated protein antibody status (Extended Data Fig. 2a,c,d).

Starting with length-scaled transcripts per million (TPM) counts derived using the R package tximport, Limma voom was used for normalization of data and calculation of weights for linear modeling^48^.

### Cluster analysis

For cluster analysis, after removal of low-expressed genes, VST data were filtered using a coefficient of variation cutoff of >0.075 to select the 22,256 (of 56,809) most variable genes . These genes were used for cluster analysis of all baseline patients (*n* = 133) using the M3C algorithm^11^ with partitioning around medoids clustering and 1,000 iterations. The lowest penalized cluster stability Index was used to select the number of clusters. After cluster assignment, patients were split into treatment groups using Pearson’s distance metric and complete linkage method, and plotted using the ComplexHeatmap package (v.2.2.0) in R. An *χ*^2^ test was applied to test significance between clusters and response to treatment based on the trial primary outcome measure (CDAI 50%) and, additionally, EULAR C-reactive protein (CRP) response (EULAR response) as another commonly used criterion.

### Differential expression and modular analysis of RNA-seq data

Samples from all patients treated with either rituximab or tocilizumab throughout the trial were included in DEG analysis. This also comprised nonresponders who, as per trial protocol, were switched to the alternative medication at week 16, as shown in Supplementary Fig. 1. Neither responders nor nonresponders showed any significant differences in their baseline characteristics, including histological and molecular B cell status, gender or disease duration (Supplementary Table 6). Low-expressed genes were excluded from analysis, with the remaining 30,841 used for DEG analysis. This was based on negative binomial distribution via regression models of normalized count data using DESeq2, and a Wald test to compare variation between treatment response groups in synovium RNA-seq samples. Wald test-derived *P* values were FDR adjusted using Storey’s *q*-value, with a cutoff of *q* < 0.05 used to define significantly DEGs (Supplementary Data 1). Distributions of DEGs are illustrated in volcano plots, and DESeq2 outputs were used for further modular analysis with the Bioconductor package QuSAGE v.2.10.0. Gene modules from Li et al.^12^ and WGCNA modules were selected for gene set enrichment.

### Deconvolution

MCP-counter^10^ was used to deconvolute synovial RNA-seq, with the package Immunedeconv. Following deconvolution, patients were classified into rich/poor according to the median value of the individual cell type (for example, B cell rich if above the median value of MCP B cells, poor if below). For the enrichment of four fibroblast subtypes (SC-F1: *CD34*^+^ *sublining*, SC-F2: *HLA*^+^ *sublining*, SC-F3: *DKK3*^+^ *sublining* and SC-F4: *CD55*^+^ *lining*), we used average expression of gene signatures obtained from differential gene expression analysis and known markers previously described by scRNA-seq^14^. Module scores for each subtype were calculated using the AddModuleScore function in the R package Seurat. The top five differentially expressed genes were considered subtype-specific gene sets and did not have genes in common. Wilcoxon testing was used for statistical assessment of module scores when comparing responders and nonresponders.

### Crossover analysis of patients who underwent treatment switch

The drug-crossover analysis was performed on baseline RNA-seq samples of patients who underwent treatment switch (Fig. 3a). RNA-seq counts of protein-coding genes (*n* = 19,508) were used to perform a likelihood ratio test (LRT) that was calculated in comparison to a reduced model with the DESeq2 R package. Three-dimensional volcano plots and radial plots were generated using the volcano3D (v.1.0.3) package in R (Fig. 3c–e). QuSAGE was applied using WGCNA-derived gene modules, and radial plots were created using the volcano3D package with a *P* value significance threshold of <0.05 (Fig. 3e).

### Multiplex immunofluorescence

Immunofluorescence staining was performed on 3-µm, formalin-fixed, paraffin-embedded human sections obtained from synovial tissues of patients with RA. Tissue sections were deparaffinized in sequential changes of xylene and ethanol chambers before washing and placing in a preheated target retrieval solution (pH 6.0; Dako, no. S1699) in a pressure cooker for 15 min. Tissue sections were allowed to cool at room temperature (RT) before washing in Tris-buffered saline (TBS). Endogenous peroxidase and biotin activity were blocked with peroxidase (Dako, no. S2023) for 10 min at RT.

Antibody specifications used for immunofluorescence can be found in Supplementary Table 7. In brief, for CD90/CD45/DKK3 staining, protein block (Dako, no. X0909) was applied for 1 h, slides were stained with the first primary antibody (CD45; Dako, no. M0701, mouse IgG1), washed three times in TBS then incubated with Anti-Mouse Envision system horseradish peroxidase (HRP; Dako, no. K4001) for 30 min at RT. After three washes in TBS, the Cy5/Alx647-conjugated Tyramide reagent (1:100; Thermofisher, no. B40958) was applied for 3 min. After three washes in TBS, antibody stripping was performed by placing slides in preheated target retrieval solution (pH 6.0; Dako, no. S1699) in a pressure cooker for 15 min. This process was repeated for one of two additional primary antibodies: CD90 (1:240; Abcam, no. 133350, rabbit) or DKK3 (1:150; Sigma-Aldrich, no. HPA011868, rabbit), followed by Anti-Rabbit Envision system HRP (Dako, no. K4003) and Alx488-conjugated Tyramide reagent for CD90 (1:100; Thermofisher, no. B40953) or Alx555-conjugated Tyramide reagent for DKK3 (1:100; Thermofisher, no. B40955), with antibody stripping in between as described above.

DAPI (Thermofisher) nuclear counterstaining was applied for 10 min at RT and slides were then mounted with ProLong Gold Antifade reagent (Thermofisher).

Images were captured using a NanoZoomer S60 Digital slide scanner (Hamamatsu, no. C13210-01) at ×20 magnification at a resolution of 440 nm per pixel (DPI, no. 57727), with the following exposure times: CD45 alx647 Cy5, 16 ms; CD90 alx488 FITC, 32 ms; DKK3 alx555 TRITC, 24 ms; DAPI, 224 ms. Image analysis was performed using NDP.view 2 Software (Hamamatsu Photonics, no. U12388-01).

### GeoMx DSP

Formalin-fixed, paraffin-embedded synovial tissue from 12 patients with RA, before treatment with rituximab or tocilizumab, was profiled using the GeoMx DSP platform as previously described^49^. Briefly, tissue morphology was visualized using fluorescent antibodies CD68-AF532 (clone KP1, Novus), CD20-DL594 (clone IGEL/773, Novus) and CD3-AF647 (clone UMAB54, Origene) and Syto13 (ThermoFisher).

For the NanoString GeoMx DSP WTA assay, slides were prepared following the automated Leica Bond RNA Slide Preparation Protocol (NanoString, no. MAN-10131-03). In situ hybridizations with the GeoMx Whole Transcriptome Atlas Panel (WTA, 18,677 genes) at 4-nM final concentration were done in Buffer R (NanoString). Morphology markers were prepared for four slides concurrently using Syto13 (DNA), CD20, CD3 and CD68 in Buffer W for a total volume of 125 μl per slide. Slides incubated with 125 μl of morphology marker solution at RT for 1 h, then washed in SSC and loaded onto the NanoString DSP instrument.

On the DSP instrument each slide was scanned with a ×20 objective at scan parameters 60 ms FITC/525 nm, 200 ms Cy3/568 nm, 250 ms Texas Red/615 nm and 300 ms Cy5/666 nm.

The resulting immunofluorescent images were used to select six freeform polygon-shaped ROIs containing approximately 200 nuclei in CD68^+^ synovial tissue lining and superficial sublining, CD20^–^CD3^–^ sublining and CD20^+^CD3^+^ lymphocyte aggregates.

After approval of ROIs, GeoMx DSP photocleaved the ultraviolet (UV)-cleavable barcoded linker of bound RNA probes and collected individual segmented areas into separate wells in a 96-well collection plate.

The dataset included 72 ROIs from 12 patients (four refractory and eight responder) across the three ROI types. An NTC water well was used for quality control checks.

### DSP analysis

GeoMx WTA sequencing reads from NovaSeq6000 were compiled into FASTQ files corresponding to each ROI. FASTQ files were then converted to digital count conversion files using the NanoString GeoMx NGS DnD Pipeline. Out of 18,677 genes, 17,065 exceeded the lower level of quantitation (LOQ) in >10% of ROIs; genes that did not exceed LOQ were excluded from the analysis. For normalization, counts were divided by sample-specific size factors determined by the median ratio of gene counts relative to geometric mean per gene. The DESeq2 R package was used for this preprocessing step.

### Differential expression analysis

We conducted differential expression analysis to compare responders and refractory patients using DESeq2^50^. This analysis was done for all ROIs simultaneously (responders, *n* = 48; refractory, *n* = 24) but separately for each location in the synovial layer: CD68^+^ lining/superficial sublining (responders, *n* = 17; refractory, *n* = 8); CD20^–^CD3^–^ deep sublining (responders, *n* = 21, refractory *n* = 12); and CD3^+^CD20^+^ lymphoid aggregates (responders, *n* = 10; refractory, *n* = 4). Since samples were collected from different locations, in the analysis of all samples we included location as a covariate (~location + response) to eliminate its influence on gene expression. The *qvalue* R package implementing Storey’s *q*-value method was used to correct for multiple testing effects, and a cutoff of *q* < 0.05 was used to define significantly DEGs.

### Longitudinal mixed-effects model analysis

Longitudinal analysis of RNA-seq on paired synovial biopsies was performed by fitting a negative binomial distribution GLMM for each gene. Because the most widely used mainstream differential gene expression analysis tools—edgeR, DESeq2^47^ and Limma voom^48^—are all unable to fit mixed-effects linear models, we developed the R package glmmSeq to fit negative binomial mixed-effects models at the individual gene level. glmmSeq uses the glmer function from the R package lme4 (v.1.1-25), with negative binomial family function from the MASS package (v.7.3-53). Models were fit using maximum-likelihood estimation by Laplace approximation and bound optimization by quadratic approximation. For analysis of the differential effects of the two trial medications over time, the following model was fitted for each gene individually:

$$\begin{array}{c}{{Y}}_{{{{\mathrm{ijg}}}}}\sim {{{\mathrm{NB}}}}\left( {\mu _{{{{\mathrm{ijg}}}}},\alpha _{{{\mathrm{g}}}}} \right)\\ {{{\mathrm{log}}}}\left( {\mu _{{\mathrm{ijg}}}} \right) = o_{{\mathrm{ij}}} + \beta _{{\mathrm{g}}0} + \beta _{{\mathrm{g}}1}{{{\mathrm{time}}}}_{{{{\mathrm{ij}}}}} + \beta _{{\mathrm{g}}2}{{{\mathrm{medication}}}}_{{{{{\mathrm{i}}}}}} + \beta _{{{{\mathrm{g}}}}3}{{{\mathrm{time}}}}_{{{{\mathrm{ij}}}}}{{{\mathrm{medication}}}}_{\mathrm{i}} + {{{{b}}}}_{{{{\mathrm{gi}}}}}\\ {{{{b}}}}_{{{{\mathrm{gi}}}}}\sim N\left( {0,\sigma _{{{{\mathrm{gb}}}}}^2} \right)\end{array}$$

where *Y*_ijg_ is the longitudinal raw count of gene g in individual I at timepoint j, *α*_g_ is the dispersion parameter for each gene, *o*_ij_ is an offset term scaled to the logarithm of the total library size for each sample, *b*_gi_ are random effects between individual patients, and N and NB are the functions for normal distribution and negative binomial distribution, respectively. TPM counts were used as input, and only individuals with paired samples were included (88 samples, 44 individuals). The dispersion parameter for the negative binomial distribution for each gene was calculated using the DESeq2 function estimateDispersions. To reduce the problem of inflated model coefficients relating to zero counts, genes of low expression were removed using the Limma (v.3.44.3) function filterByExpr and zero counts were adjusted to a pseudo-count of 0.125, equivalent to the ‘prior count’ approach of edgeR and Voom^48^ whose internal defaults are 0.125 and 0.5, respectively. Statistical testing of the fitted model coefficients was performed using the Wald type 2 *χ*^2^ test from the car package (v.3.0-10). *P* values were FDR adjusted using Storey’s *q*-value, with a cutoff of FDR < 0.05 considered significant for each term in the model (Supplementary Data 4). Predictions were calculated for each fitted gene model based on the fitted linear model coefficients, and 95% CIs for the fixed effects of the fitted model were calculated from standard deviations of the predictions by extracting prediction variances as the diagonal from the variance–covariance matrix of the predictions *XVX*, where *X* represents the model matrix corresponding to the new data and *V* is the variance–covariance matrix of the model parameters. Similarly, for analysis of the difference between CDAI 50% responders and nonresponders following drug exposure for each medication, the following model was fitted for each drug cohort (58 samples, 29 individuals for rituximab; 30 samples, 15 individuals for tocilizumab):

$${{{\mathrm{log}}}}\left( {\mu _{{{{\mathrm{ijg}}}}}} \right) = o_{{{{\mathrm{ij}}}}} + \beta _{{{{\mathrm{g}}}}0} + \beta _{{{{\mathrm{g}}}}1}{{{\mathrm{time}}}}_{{{{\mathrm{ij}}}}} + \beta _{{{{\mathrm{g}}}}2}{{{\mathrm{response}}}}_{{{\mathrm{i}}}} + \beta _{{{{\mathrm{g}}}}3}{{{\mathrm{time}}}}_{{{{\mathrm{ij}}}}}{{{\mathrm{response}}}}_{{{\mathrm{i}}}} + {{{{b}}}}_{{{{\mathrm{gi}}}}}$$

The R package glmmSeq is downloadable via CRAN and the source code is also available, from https://github.com/KatrionaGoldmann/glmmSeq. When compared against a Gaussian linear mixed-effects model on log count data, glmmSeq showed similar results with strong correlation between *P* values generated using either distribution (Supplementary Fig. 2a–c). Q–Q plots suggested that the negative binomial mixed model showed greater power in identification of significant effects (Supplementary Fig. 2d–f).

### Longitudinal pathway analysis

Genes showing a significant change in the analysis described in the previous section were used for GO/pathway enrichment analysis by means of the clueGO (v.2.5.5) Cytoscape plug-in. To allow an automated enrichment process, clueGO REST-enabled features were used in R using the following GO/pathway repositories: *BiologicalProcess-EBI-UniProt-GOA (11 February 2020)*, *CellularComponent-EBI-UniProt-GOA (11 February 2020)*, *ImmuneSystemProcess-EBI-UniProt-GOA (11 February 2020)*, *MolecularFunction-EBI-UniProt-GOA (11 February 2020)*, *KEGG (27 February 2019)* and *REACTOME (27 February 2019)*.

### Building classifier models for prediction of rituximab and tocilizumab response and refractory status

Baseline gene expression and clinical and histological data were used as features for machine learning models built to predict CDAI 50% response to either rituximab or tocilizumab treatment at the primary endpoint (16 weeks) or refractory status, defined as nonresponse to both drugs at the secondary endpoint (post-treatment crossover, 24 weeks). An overview of the pipeline is shown in Fig. 6a.

Although the R4RA study was not powered for machine learning, information theory demonstrates that sparse models developed from large biomarker panels in which only a small percentage of biomarkers have nonzero effects can still demonstrate evidence of prediction with relatively modest sample size, although the small sample sizes in our study mean that the predictive models are likely to be only part of the way up the learning curve^41^. In the present study ~2% of the 1,500 biomarkers inputted into the modeling system have a nonzero effect. If the *C*-statistic of the optimal classifier is 0.83, a sample size equivalent to 0.05 events per variable (*n* = 83) would be required to learn a classifier that has expected information for discrimination equal to 25% of that obtained by the optimal classifier (equivalent to a *C*-statistic of 0.68) (see the online calculator, https://pmckeigue.shinyapps.io/sampsizeapp/)^41^. The model feature space was created using either clinical and histological parameters or clinical data with gene expression. Gene expression data underwent VST and were subset to protein-coding genes (using gencode gene annotation v.29) with the highest expression variance (top 10%). Highly correlated genes (*r* > 0.9) were removed using the *findCorrelation* function from the R package caret (v.6.0-86), leaving 1,438 genes. Clinical features included: baseline tender joint count (TJC), swollen joint count (SJC), age, gender, CDAI, erythrocyte sedimentation rate (ESR), CRP and disease activity score based on ESR and CRP (DAS28ESR and DAS28CRP, respectively). Histology features included CD3, CD68L, CD68SL, CD20 and CD138.

Following processing, data were split into 10 × 10 nested folds (Fig. 6a (2)). For models using gene expression features, filtering was performed using either RFE or univariate filtering from the *caret* package v.6.0. The number of features selected was chosen to maximize accuracy from 25, 30, 50 or 100. Model hyperparameters were tuned by inner tenfold cross-validation, with model accuracy determined in separate outer cross-validation folds to give an unbiased estimate of model accuracy.

Seven machine learning methodologies from the *caret* package were used to create the classifier models: elastic net (glmnet), random forest (RF), least-squares support vector machine (SVM) with radial basis function kernel (svmRadial), least-squares SVM with polynomial kernel (svmPoly), GBM, MDA and PDA. Models that failed to converge during training were excluded from evaluation. The purpose of testing multiple models was to determine whether nonlinear decision boundaries—as used by SVM, MDA, PDA and tree-based prediction algorithms such as GBM—could outperform penalized linear regression.

To evaluate model performance, receiver operating characteristic (ROC) curves were built using the *plotROC* R package v.2.2.1 to determine prediction accuracy in the outer fold test data and samples omitted for the inner fold. AUC was calculated to determine prediction performance. Tuning parameters for the final model were finalized as the mean over all ten outer folds. The final best model for each classification was fit to the entire dataset, exported and feature importance ranked.

### Statistical analysis

For cross-sectional comparisons of continuous variables between two groups the Mann–Whitney *U*-test was used, whereas the Wilcoxon signed-rank test was used to assess the difference between groups with longitudinal paired data. More specific analyses of RNA-seq count data are detailed above in each relevant section. R v.4.0.0, or later, was used for all formal testing analyses.

### Reporting Summary

Further information on research design is available in the Nature Research Reporting Summary linked to this article.

## Online content

Any methods, additional references, Nature Research reporting summaries, source data, extended data, supplementary information, acknowledgements, peer review information; details of author contributions and competing interests; and statements of data and code availability are available at 10.1038/s41591-022-01789-0.

### Supplementary information

Supplementary Information Supplementary Figs. 1 and 2, Tables 1–7 and a list of R4RA collaborative group members.

Reporting Summary

Supplementary Data 1 Synovial biopsy DEGs in rituximab and tocilizumab responders compared with nonresponders.

Supplementary Data 2 Three-way DEG analysis of patients who were pro-rituximab, pro-tocilizumab or refractory.

Supplementary Data 3 Modular analysis of patients who were pro-rituximab, pro-tocilizumab or refractory.

Supplementary Data 4 Longitudinal mixed-model gene expression analysis.

Supplementary Data 5 R objects for optimal prediction models for rituximab (glmnet), tocilizumab (glmnet) and refractory state (GBM), including feature space data for ten dummy samples and R script outlining model interrogation and use for future predictions.

## Figures and Tables

**Extended Data Table 1:**
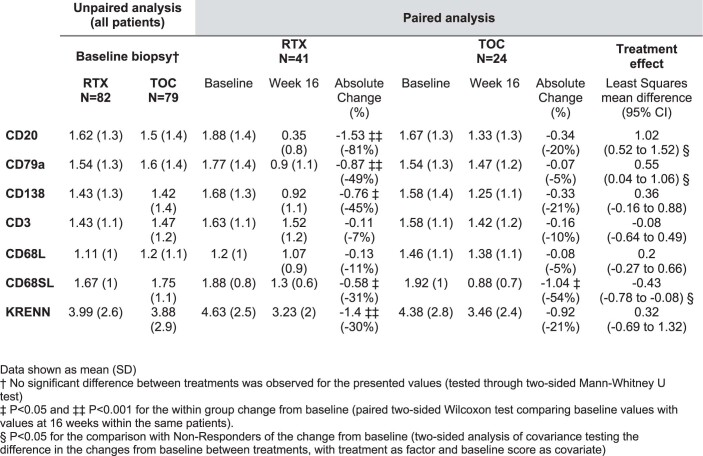
Synovial histological analysis stratified according to treatment at baseline and 16 weeks

**Extended Data Table 2:**
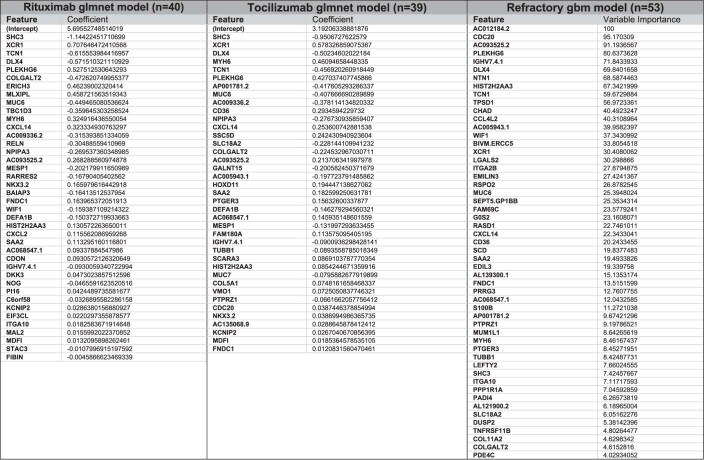
Genes included in prediction models

## Data Availability

The datasets generated during and/or analyzed during the current study are available on an interactive web interface that allows direct data exploration (https://r4ra.hpc.qmul.ac.uk/). A searchable interface is available to examine relationships between individual synovial gene transcript levels and histological and clinical parameters, and clinical response at 16 weeks. In addition, interactive versions of Figs. 3c and 5b and Extended Data Fig. 5e,f allow users to click on individual genes to see their expression and search for genes of interest. The website was constructed using R shiny server 1.5.16, with interactive plots generated with R plotly 4.9.3. The datasets can be downloaded from https://www.ebi.ac.uk/arrayexpress/experiments/E-MTAB-11611. Other public datasets used for pathway analysis were sourced from the GO annotation (GOA) database (BiologicalProcess-EBI-UniProt-GOA (11 February 2020), CellularComponent-EBI-UniProt-GOA (11 February 2020), ImmuneSystemProcess-EBI-UniProt-GOA (11 February2020), MolecularFunction-EBI-UniProt-GOA (11 February 2020)), KEGG and Reactome.

## Code availability

Source code for all analyses written in R v.4.0.0 or later has been uploaded to Github and is available at https://github.com/EMR-bioinformatics/R4RA. The R package glmmSeq is downloadable via CRAN, and the source code is available from https://github.com/KatrionaGoldmann/glmmSeq. R objects for all classifiers are available in the supplementary material as Supplementary Data 5.
