# Supplementary material for: Rituximab versus tocilizumab in rheumatoid arthritis: synovial biopsy-based biomarker analysis of the phase 4 R4RA randomized trial
Source: Nat Med. 2022 May 19;28(6):1256–68. doi: 10.1038/s41591-022-01789-0 (PMC9205785; doi:10.1038/s41591-022-01789-0)
Supplement: Supplementary file 1 — Supplementary Figs. 1 and 2, Tables 1–7 and a list of R4RA collaborative group members. [file 41591_2022_1789_MOESM1_ESM.pdf]

nature portfolio

https://doi.org/10.1038/s41591-022-01789-0

Supplementary information

Rituximab versus tocilizumab in rheumatoid arthritis: synovial biopsy-based biomarker analysis of the phase 4 R4RA randomized trial

In the format provided by the authors and unedited

Supplementary Figure S1 | Trial scheme

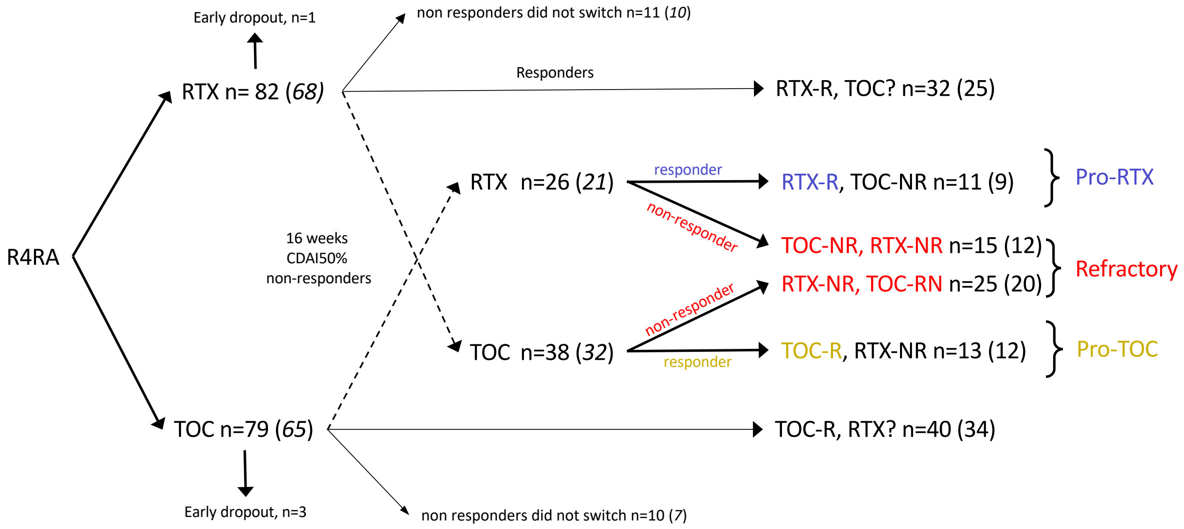

Tocilizumab (TOC), rituximab (RTX), responder (R), non-responder (NR), n = samples available for histology, (n) = samples available for RNAseq

Supplementary Figure 2 | Comparison of negative binomial mixed effects model and gaussian mixed effects model applied to log count data

a, Correlation plots of –log10 P values for analysis comparing effect of each drug over time using model gene ~ drug * time + (1 | patient). Gaussian p values for model parameters time, drug and interaction term time:drug are plotted on the x axis, while negative binomial mixed effects model p values are plotted on the y axis. P values are shown as –log10. Each point represents a single gene. Red line and shaded grey region shows linear regression line with 95% confidence intervals.

b, Similar analysis comparing Gaussian mixed model and negative binomial mixed model for the comparison of responders vs non-responders over time for the rituximab treated cohort.

c, Similar analysis comparing Gaussian mixed model and negative binomial mixed model for the comparison of responders vs non-responders over time for the tocilizumab treated cohort.

d-f, QQ plots showing observed vs expected –log10 p values for each of the 3 analyses in (a-c) demonstrating substantially increased power of negative binomial mixed models compared to Gaussian models for detecting significant genes.

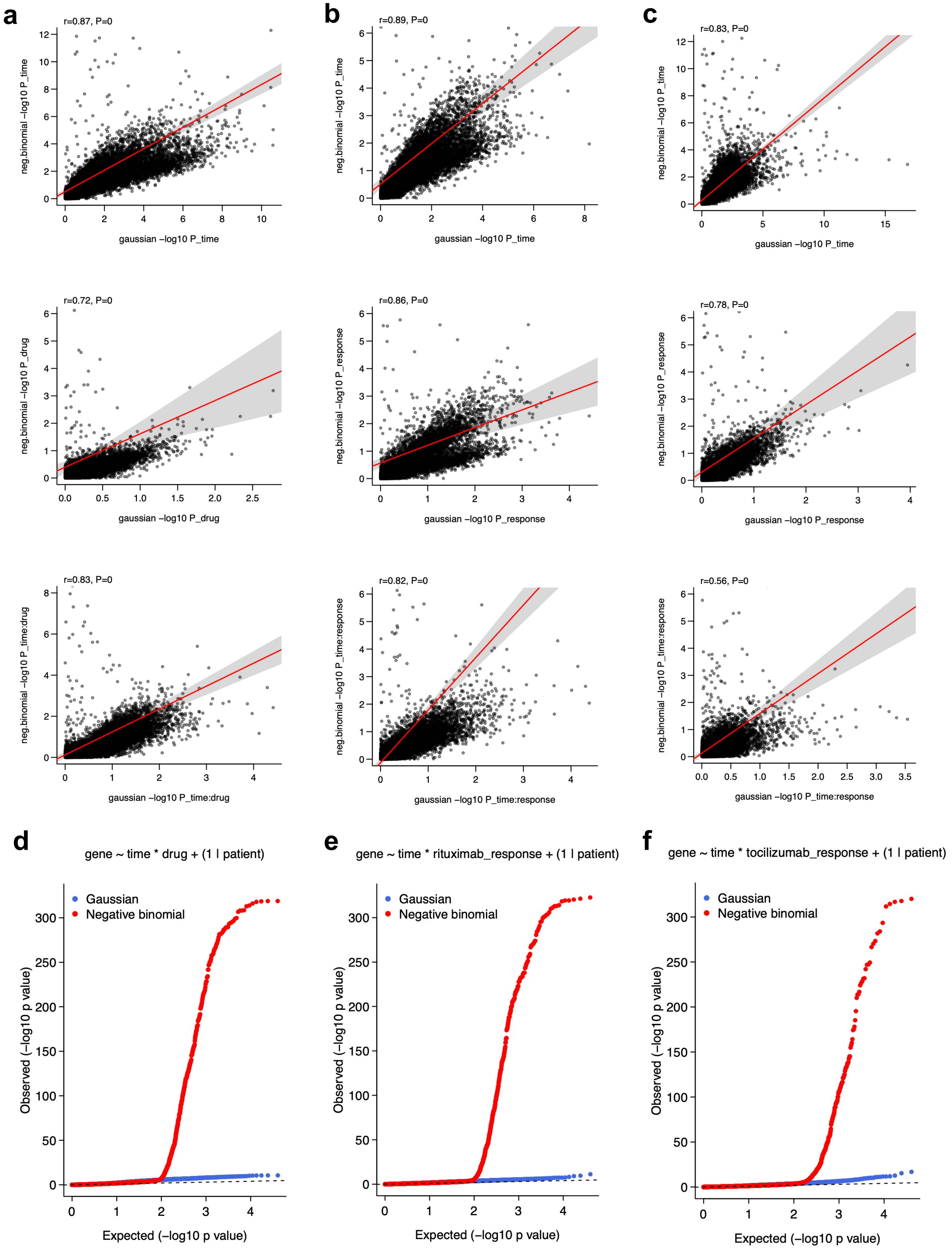
a
b
c
d
e
f
r=0.87, P=0
r=0.89, P=0
r=0.83, P=0
r=0.72, P=0
r=0.86, P=0
r=0.78, P=0
r=0.83, P=0
r=0.82, P=0
r=0.56, P=0
neg.binomial −log10 P_time
gaussian −log10 P_time
neg.binomial −log10 P_drug
gaussian −log10 P_drug
neg.binomial −log10 P_response
gaussian −log10 P_response
neg.binomial −log10 P_time:drug
gaussian −log10 P_time:drug
neg.binomial −log10 P_time:response
gaussian −log10 P_time:response
gene ~ time * drug + (1 | patient)
gene ~ time * rituximab_response + (1 | patient)
gene ~ time * tocilizumab_response + (1 | patient)
Gaussian
Negative binomial
Observed (−log10 p value)
Expected (−log10 p value)

SUPPLEMENTARY TABLES

Supplementary Table S1 | Machine learning model evaluation using nested cross-validation

Model	Filtering	AUC	Accuracy	Number of input features	Number of sparse output features	Precision	Youden sensitivity	Youden specificity
**Rituximab**								
glmnet	Univariate	0.744	0.662	117	40	0.562	0.8400	0.628
svmRadial	Univariate	0.725	0.676	134	133	0.556	0.9600	0.512
glmnet	Univariate	0.718	0.676	136	26	0.565	0.7600	0.698
svmRadial	Univariate	0.707	0.706	118	117	0.609	0.6800	0.721
gbm	Univariate	0.706	0.676	126	54	0.579	0.8000	0.558
gbm	Univariate	0.701	0.706	137	92	0.619	0.9200	0.465
svmPoly	Univariate	0.694	0.662	143	142	0.545	0.8800	0.535
svmPoly	Univariate	0.694	0.662	124	123	0.538	0.9200	0.488
rf	Univariate	0.687	0.647	125	124	0.522	0.9200	0.558
rf	Univariate	0.687	0.603	129	128	0.429	0.8800	0.535
glmnet	None	0.638	0.618	1438	31	0.471	0.7200	0.605
glmnet	None	0.624	0.632	1438	0*	0.500	0.8800	0.395
pda	Univariate	0.577	0.574	119	118	0.423	0.4400	0.767
mda	Univariate	0.532	0.544	0*	0*	0.375	0.8000	0.419
**Tocilizumab**								
glmnet	Univariate	0.681	0.615	117	39	0.655	0.6471	0.677
glmnet	None	0.648	0.585	1438	14	0.590	0.5588	0.710
gbm	Univariate	0.626	0.585	125	95	0.606	0.4118	0.806
gbm	Univariate	0.597	0.569	140	92	0.600	0.3235	0.935
glmnet	Univariate	0.582	0.600	136	45	0.618	0.6765	0.581
pda	Univariate	0.554	0.538	121	120	0.571	0.7650	0.452
mda	Univariate	0.542	0.569	0*	0*	0.575	0.9120	0.258
svmRadial	Univariate	0.524	0.462	128	127	0.484	0.7647	0.387
svmRadial	Univariate	0.503	0.523	131	130	0.552	0.5588	0.581
rf	Univariate	0.490	0.492	135	134	0.514	0.3824	0.742
svmPoly	Univariate	0.457	0.462	133	132	0.486	0.0294	1.000
svmPoly	Univariate	0.430	0.446	130	129	0.471	0.2647	0.774
rf	Univariate	0.412	0.385	133	132	0.421	0.2647	0.774
**Refractory**								
gbm	Univariate	0.686	0.696	135	53	0.750	0.762	0.562
rf	Univariate	0.683	0.723	128	127	0.758	0.700	0.656
glmnet	None	0.682	0.732	1438	14	0.798	0.762	0.562
rf	Univariate	0.678	0.732	118	117	0.772	0.500	0.844

svmPoly	Univariate	0.678	0.705	120	119	0.753	0.637	0.688
glmnet	None	0.673	0.705	1438	0*	0.747	0.838	0.438
glmnet	None	0.661	0.625	1438	43	0.711	0.650	0.688
svmRadial	Univariate	0.654	0.741	142	141	0.758	0.850	0.469
svmRadial	Univariate	0.649	0.723	134	133	0.769	0.637	0.656
rf	Univariate	0.645	0.696	142	141	0.750	0.600	0.688
svmPoly	Univariate	0.632	0.714	128	127	0.750	0.637	0.719
svmPoly	Univariate	0.592	0.723	132	131	0.753	0.863	0.406
glmnet	Univariate	0.586	0.661	137	24	0.733	0.650	0.594
glmnet	Univariate	0.567	0.652	125	29	0.730	0.537	0.719
svmRadial	Univariate	0.566	0.688	132	131	0.742	0.688	0.562
mda	Univariate	0.566	0.607	142	141	0.757	0.875	0.344
pda	Univariate	0.564	0.634	146	145	0.760	0.588	0.656
glmnet	Univariate	0.552	0.634	124	26	0.714	0.662	0.531

glmnet = lasso and elastic-net generalized linear model; rf= random forest; gbm=gradient boosting machine; svmRadial=radial support vector machine; svmPoly = polynomial support vector machine.

* Final fit failed.

Supplementary Table S2 | Best tuned parameters for final models

i) Rituximab glmnet model				ii) Tocilizumab glmnet model				iii) Refractory gbm model					
Outer fold	alpha	lambda	N of sparse features	Outer fold	alpha	lambda	N of sparse features	Outer fold	N trees	Interaction depth	shrinkage	N min obs. in node	Number of features
**Fold 1**	1	0.0143845	16	Fold 1	1	6.1585E-05	23	Fold 1	100	1	0.1	10	65
**Fold 2**	0.7	0.0143845	29	Fold 2	0.7	0.02636651	19	Fold 2	50	3	0.1	10	107
**Fold 3**	0.7	0.0078476	35	Fold 3	0.7	0.00428133	33	Fold 3	50	3	0.1	10	108
**Fold 4**	0.8	0.02636651	21	Fold 4	0.7	0.02636651	24	Fold 4	100	2	0.1	10	117
**Fold 5**	0.8	0.00428133	34	Fold 5	0.9	0.0143845	27	Fold 5	150	3	0.1	10	216
**Fold 6**	0.9	0.00428133	30	Fold 6	0.7	0.00428133	26	Fold 6	100	1	0.1	10	76
**Fold 7**	0.8	0.00428133	32	Fold 7	0.7	0.0143845	28	Fold 7	100	2	0.1	10	125
**Fold 8**	0.9	0.0143845	18	Fold 8	0.7	0.0483293	27	Fold 8	50	2	0.1	10	76
**Fold 9**	0.9	0.0078476	28	Fold 9	0.9	0.0078476	23	Fold 9	150	1	0.1	10	83
**Fold 10**	0.8	0.02636651	21	Fold 10	0.7	0.0078476	26	Fold 10	100	1	0.1	10	63
**Average**	**0.83**	**0.01244257**	**26.4**	**Average**	**0.77**	**0.01541508**	**25.6**	**Average**	**95**	**1.9**	**0.1**	**10**	**103.6**

Supplementary Table S3 |Baseline characteristics of all of patients which consented to a second biopsy at 16 weeks

	Patients which consented to a second biopsy at 16 weeks			
	Overall (n=59)	Rituximab (n=37)	Tocilizumab (n=22)	Unadjusted p value
**Gender = Male**	9 (15)	6 (16)	3 (14)	1
**Age, years**	54.2 (14.3)	54.6 (13.9)	53.6 (15.2)	0.79
**Disease Duration, years**	9.0 [4.0, 16.0]	10.0 [4.0, 17.0]	6.0 [3.2, 14.2]	0.17
**Clinical disease activity index (CDAI)**	28.9 [22.2, 38.4]	30.4 [24.5, 38.5]	27.4 [21.7, 36.4]	0.4
**Erythrocyte sedimentation rate (ESR), mm/h**	35.0 [19.5, 50.0]	38.0 [20.0, 52.0]	32.0 [20.0, 44.0]	0.46
**C-reactive protein (CRP), mg/L**	10.0 [5.0, 23.0]	10.0 [5.0, 23.0]	11.5 [7.2, 28.8]	0.38
**Rheumatoid factor (RF) OR Anti–citrullinated protein antibody (ACPA) positive**	51 (86)	35 (95)	16 (73)	0.048
**Rheumatoid factor (RF) positive**	43 (73)	30 (81)	13 (59)	0.12
**Anti–citrullinated protein antibody (ACPA) positive**	49 (83)	33 (89)	16 (73)	0.2
**Creatinine (μmol/L)**	57.0 [50.0, 66.0]	56.5 [51.5, 67.5]	57.0 [46.0, 63.0]	0.65
**Alanine aminotransferase (ALT), U/L**	16.0 [11.0, 20.0]	14.0 [11.0, 18.5]	16.5 [13.5, 23.2]	0.12
**Aspartate aminotransferase (AST), U/L**	19.0 [16.0, 23.0]	19.0 [15.5, 23.0]	17.0 [16.0, 23.0]	0.84
**Haemoglobin, g/L**	119.0 (14.5)	117.0 (13.7)	122.4 (15.6)	0.18
**White Blood Cell count, 10^9/L**	8.7 (2.7)	8.1 (2.7)	9.5 (2.6)	0.061
**Platelets, 10^9/L**	310.0 [263.0, 388.0]	311.0 [279.2, 359.5]	283.0 [255.0, 388.0]	0.54
**Neutrophils, 10^9/L**	6.2 (2.4)	5.9 (2.3)	6.7 (2.4)	0.26
**Lymphocytes, 10^9/L**	1.6 [1.2, 2.2]	1.4 [1.1, 2.1]	1.7 [1.5, 2.3]	0.023
**Synovial semi-quantitative scores**				
CD20	2.0 [1.0, 3.0]	2.0 [1.0, 3.0]	2.0 [1.0, 3.0]	0.72
CD138	2.0 [0.0, 3.0]	2.0 [0.0, 3.0]	1.5 [1.0, 3.0]	0.92
CD68 lining	1.0 [1.0, 2.0]	1.0 [1.0, 2.0]	1.0 [1.0, 2.0]	0.42
CD68 sub-lining	2.0 [1.0, 3.0]	2.0 [1.0, 3.0]	2.0 [1.2, 3.0]	0.5
CD3	2.0 [1.0, 2.5]	2.0 [1.0, 2.0]	2.0 [1.0, 2.8]	0.78
CD21 (Positive)	5 (9)	4 (11)	1 (5)	0.76
**B cell status**				0.66
B cell poor	24 (41)	14 (38)	10 (45)	
B cell rich	30 (51)	19 (51)	11 (50)	
Germinal Centre	5 (8)	4 (11)	1 (5)	
Unknown	0 (0%)	0 (0%)	0 (0%)	

**Synovial Pathotype**				0.58
Fibroid	5 (8)	4 (11)	1 (5)	
Lymphoid	41 (69)	26 (70)	15 (68)	
Myeloid	13 (22)	7 (19)	6 (27)	
Ungraded	0 (0%)	0 (0%)	0 (0%)	
**Number of tender joints, 0-28**	11.0 [7.0, 17.0]	11.0 [7.0, 19.0]	10.0 [7.0, 13.8]	0.32
**Number of swollen joints, 0-28**	6.0 [4.0, 8.5]	6.0 [4.0, 8.0]	4.0 [3.2, 10.0]	0.62
**28 joint count Disease Activity Score (DAS-28), ESR**	5.9 (1.1)	5.9 (1.1)	5.8 (1.1)	0.64
**28 joint count Disease Activity Score (DAS-28), CRP**	5.3 (1.0)	5.3 (1.0)	5.3 (1.1)	0.85
**Patient's global assessment, 0–100 VAS**	70.0 [51.0, 79.0]	64.0 [50.0, 78.0]	72.0 [52.8, 85.5]	0.22
**Physician's global assessment, 0–100 VAS**	59.6 (20.8)	60.0 (19.7)	59.0 (23.0)	0.85
**Patient's assessment of early morning stiffness, 0–100 VAS**	52.5 [20.0, 100.0]	34.0 [20.0, 70.0]	60.0 [22.5, 100.0]	0.34
**Patient's assessment of tiredness, 0–100 VAS**	67.0 [52.0, 81.0]	67.0 [52.0, 73.0]	71.0 [52.0, 93.5]	0.24
**Patient's assessment of pain, 0–100 VAS**	66.0 [48.5, 84.0]	65.0 [52.0, 78.0]	73.0 [41.8, 89.5]	0.39
**HAQ total score**	1.7 (0.6)	1.7 (0.7)	1.7 (0.5)	0.92
**Functional Assessment of Chronic Illness Therapy (FACIT) score**	21.4 (10.7)	21.1 (11.1)	21.9 (10.1)	0.78
**Previous Methotrexate use**	59 (100)	37 (100)	22 (100)	
**Previous Prednisolone use**	29 (49)	18 (49)	11 (50)	1
**Number of previous biologics used, [anti-TNF/Other**]**				0.33
1	47 (80) [47/0]	31 (84) [31/0]	16 (73) [16/0]	
2	11 (19) [10/1]	5 (14) [4/1]	6 (27) [6/0]	
3+	1 (2) [1/0]	1 (3) [1/0]	0 (0) [0/0]	

Data are n (%), median [IQR], mean (SD%). ECOG=Eastern Cooperative Oncology Group. BMI=body-mass index. CDAI=Clinical disease activity index. DAS28 =28 joint count disease activity score. CRP=C-reactive protein. ESR=erythrocyte sedimentation rate.
**6 patients in total used non-TNFi biologics (5 Abatacept and 1 "vaccine RA TNF-K-006" for a clinical study%).

Supplementary Table S4 | Demographics and disease activity of patients undergoing paired week 16 biopsy

Baseline characteristics	Total cohort (N=161)	Consent to a second biopsy No (N=96)	Yes (N=65)	Unadjusted p-value
**Gender = M**	33 (20.5)	23 (24.0)	10 (15.4)	0.261
**Age (years)**	55.7 (12.9)	56.4 (12.4)	54.6 (13.7)	0.381
**Disease Duration (years)**	9.0 [4.0, 19.0]	9.0 [5.0, 21.0]	10.0 [4.0, 16.0]	0.357
**CDAI**	29.8 [21.7, 40.6]	29.4 [21.2, 41.8]	30.4 [24.5, 39.7]	0.707
**ESR (mm/h)**	31.5 [17.0, 48.0]	28.0 [17.0, 48.0]	35.0 [19.0, 50.0]	0.202
**CRP (mg/L)**	11.0 [5.0, 27.5]	14.4 [5.0, 32.2]	10.0 [5.0, 25.0]	0.462
**RF positive**	105 (67.3)	60 (65.2)	45 (70.3)	0.621
**ACPA positive**	119 (76.8)	70 (76.9)	49 (76.6)	1.000
**Tender Joint Count (28)**	11.0 [6.0, 18.0]	10.5 [5.8, 17.0]	11.0 [7.0, 18.0]	0.248
**Swollen Joint Count (28)**	6.0 [3.0, 10.0]	6.0 [3.0, 11.2]	6.0 [4.0, 9.0]	0.919
**DAS28 (ESR)**	5.8 (1.2)	5.7 (1.4)	5.9 (1.0)	0.311
**DAS28 (CRP)**	5.3 (1.2)	5.3 (1.3)	5.3 (1.0)	0.829
**Week 16 Treatment response, N (%)**				
**CDAI ≥50% Response**	81 (50.3)	56 (58.3)	25 (38.5)	0.016*
**CDAI-MTR**	52 (32.3)	40 (41.7)	12 (18.5)	0.002*
**Baseline histological score, mean (SD)**				
**CD20+ B cells**	1.56 (1.3)	1.38 (1.3)	1.8 (1.3)	0.052
**CD138+ plasma cells**	1.43 (1.4)	1.26 (1.4)	1.65 (1.4)	0.077
**CD68L+ macrophages**	1.15 (1)	1.05 (1)	1.29 (1.1)	0.141
**CD68SL+ macrophages**	1.71 (1)	1.57 (1.1)	1.89 (0.9)	0.04*
**CD3+ T cells**	1.45 (1.2)	1.32 (1.2)	1.62 (1.1)	0.081
**Synovitis score**	3.93 (2.8)	3.48 (2.8)	4.54 (2.6)	0.019*

Data are n (%), median [IQR], mean (SD). ECOG=Eastern Cooperative Oncology Group. BMI=body-mass index. CDAI=Clinical disease activity index. DAS28 =28 joint count disease activity score. CRP=C-reactive protein. ESR=erythrocyte sedimentation rate. All comparisons were two-sided; Student's T-test or Mann-Whitney-U test was used for continuous variables

(based on normality of distribution) and Chi-Squared test or Fisher's exact test was used for categorical variables (based on low cell count occurrence, if at least one cell count in the 2xn contingency table was equal or less than 5, Fisher's test was used). No adjustment for multiple comparison was performed.

Supplementary Table S5 | Baseline characteristics of patients with available RNAseq

	Overall (n=133)	Rituximab (n=68)	Tocilizumab (n=65)	Unadjusted p value
Gender (Male)	24 (18%)	16 (24%)	8 (12%)	0·14
Age, years	55·1 (13·3)	54·7 (13·7)	55·6 (13·0)	0·69
Disease Duration, years	9·0 [5·0, 19·0]	8·0 [4·0, 21·0]	10·0 [5·0, 18·0]	0·85
Clinical disease activity index (CDAI)	30·9 [21·7, 40·7]	31·0 [22·3, 41·1]	30·4 [21·7, 40·6]	0·83
Erythrocyte sedimentation rate (ESR), mm/h	32·0 [18·0, 46·0]	34·0 [17·8, 46·2]	28·0 [19·0, 45·0]	0·62
C-reactive protein (CRP), mg/L	11·0 [5·0, 23·0]	10·0 [5·0, 20·8]	15·0 [6·0, 29·0]	0·19
Rheumatoid factor (RF) OR Anti–citrullinated protein antibody (ACPA) positive	115 (86%)	59 (87%)	56 (86%)	1
Rheumatoid factor (RF) positive	96 (72%)	52 (76%)	44 (68%)	0·35
Anti–citrullinated protein antibody (ACPA) positive	104 (78%)	54 (79%)	50 (77%)	0·89
Creatinine (µmol/L)	61·0 [54·0, 71·0]	63·0 [54·0, 72·5]	58·7 [54·0, 67·2]	0·36
Alanine aminotransferase (ALT), U/L	16·0 [11·0, 22·0]	16·0 [11·0, 20·0]	16·0 [12·0, 23·0]	0·41
Aspartate aminotransferase (AST), U/L	19·0 [15·0, 22·2]	19·0 [15·0, 22·0]	19·0 [16·0, 22·5]	0·73
Haemoglobin, g/L	121·0 [110·0, 130·5]	120·0 [108·5, 131·5]	121·5 [111·0, 130·0]	0·81
White Blood Cell count, 10^9/L	8·0 [6·7, 10·2]	7·9 [6·2, 9·7]	8·5 [7·0, 10·3]	0·17
Platelets, 10^9/L	304·0 [254·5, 394·0]	303·0 [253·0, 364·5]	304·0 [255·8, 413·8]	0·33
Neutrophils, 10^9/L	5·5 [4·4, 7·2]	5·7 [4·2, 7·1]	5·4 [4·6, 7·2]	0·28
Lymphocytes, 10^9/L	1·7 [1·3, 2·3]	1·6 [1·2, 2·2]	1·7 [1·4, 2·4]	0·15
Synovial semi-quantitative scores				
CD20	1·0 [0·0, 3·0]	1·5 [0·0, 3·0]	1·0 [0·0, 3·0]	0·85
CD138	1·0 [0·0, 3·0]	1·0 [0·0, 3·0]	1·0 [0·0, 3·0]	0·84
CD68 lining	1·0 [0·0, 2·0]	1·0 [0·8, 2·0]	1·0 [0·0, 2·0]	0·52
CD68 sub-lining	2·0 [1·0, 2·0]	2·0 [1·0, 2·0]	2·0 [1·0, 3·0]	0·38
CD3	1·0 [0·0, 2·0]	1·0 [1·0, 2·0]	2·0 [0·0, 3·0]	0·61
CD21 (Positive)	9 (8%)	5 (9%)	4 (7%)	1
Synovial Pathotype				0·22

Fibroid	24 (18%)	11 (16%)	13 (20%)	
Lymphoid	74 (56%)	36 (53%)	38 (58%)	
Myeloid	31 (23%)	17 (25%)	14 (22%)	
Ungraded	4 (3%)	4 (6%)	0 (0%)	
B cell status				0·25
B cell poor	65 (49%)	32 (47%)	33 (51%)	
B cell rich	55 (41%)	27 (40%)	28 (43%)	
Germinal Centre	9 (7%)	5 (7%)	4 (6%)	
Unknown	4 (3%)	4 (6%)	0 (0%)	
Number of tender joints, 0-28	12·0 [6·0, 18·0]	11·0 [7·0, 18·2]	12·0 [6·0, 16·0]	0·67
Number of swollen joints, 0-28	6·0 [4·0, 10·0]	6·0 [4·0, 9·0]	6·0 [3·0, 11·0]	0·73
28 joint count Disease Activity Score (DAS-28), ESR	5·8 (1·2)	5·9 (1·2)	5·8 (1·3)	0·95
28 joint count Disease Activity Score (DAS-28), CRP	5·4 (1·2)	5·3 (1·1)	5·4 (1·2)	0·62
Patient's global assessment, 0–100 VAS	72·0 [51·0, 85·0]	69·5 [48·5, 80·5]	74·0 [53·0, 86·0]	0·18
Physician's global assessment, 0–100 VAS	60·3 (21·7)	60·4 (21·3)	60·2 (22·2)	0·96
Patient's assessment of early morning stiffness, 0–100 VAS	32·5 [20·0, 100·0]	30·0 [17·5, 80·0]	45·0 [20·0, 100·0]	0·26
Patient's assessment of tiredness, 0–100 VAS	68·0 [50·0, 83·0]	67·0 [45·5, 78·2]	70·0 [50·0, 91·0]	0·17
Patient's assessment of pain, 0–100 VAS	71·0 [50·0, 87·0]	67·5 [49·8, 82·5]	73·0 [50·0, 89·0]	0·34
HAQ total score	1·7 (0·6)	1·7 (0·7)	1·7 (0·6)	0·72
Functional Assessment of Chronic Illness Therapy (FACIT) score	22·0 [15·0, 33·2]	23·0 [15·0, 32·0]	21·5 [13·0, 34·0]	0·76
Previous Methotrexate use	133 (100%)	68 (100%)	65 (100%)	
Previous Prednisolone use	74 (56%)	35 (51%)	39 (60%)	0·41
Number of previous biologics used, [anti-TNF/Other**]				0·19
1	93 (70%) [93/0]	51 (75%) [51/0]	42 (65%) [42/0]	
2	32 (24%) [29/3]	12 (18%) [9/3]	20 (31%) [20/0]	
3+	8 (6%) [5/3]	5 (7%) [3/2]	3 (5%) [2/1]	

Data are n (%), median [IQR], mean (SD%). ECOG=Eastern Cooperative Oncology Group. BMI=body-mass index. CDAI=Clinical disease activity index. DAS28 =28 joint count disease activity score. CRP=C-reactive protein. ESR=erythrocyte sedimentation rate.**6 patients in total used non-TNFi biologics (5 Abatacept and 1 "vaccine RA TNF-K-006" for a clinical study%). All comparisons were two-sided; Student's T-test or Mann-Whitney-U test was used for continuous variables (based on normality of distribution) and Chi-Squared test or Fisher's exact test was used for categorical variables (based on low cell count occurrence, if at least one cell count in the 2xn contingency table was equal or less than 5, Fisher's test was used). No adjustment for multiple comparison was performed.

Supplementary Table S6 | Baseline characteristics of patients with RNA-Seq samples, stratified by treatment and CDAI50 response

	Overall (n=133)				Rituximab (n=68)				Tocilizumab (n=65)			
	All patients (n=133)	Responders (n=67)	Non-responders (n=66)	Unadjusted p-value	All patients (n=68)	Responders (n=39)	Non-responders (n=29)	Unadjusted p-value	All patients (n=65)	Responders (n=28)	Non-responders (n=37)	Unadjusted p-value
Histology B cell status				0·665				0·611				0.53
B cell poor	65 (49%)	32 (48%)	33 (50%)		32 (47%)	19 (49%)	13 (45%)		33 (51%)	13 (46%)	20 (54%)	
B cell rich	55 (41%)	30 (45%)	25 (38%)		27 (40%)	16 (41%)	11 (38%)		28 (43%)	14 (50%)	14 (38%)	
Germinal Centre	9 (7%)	4 (6%)	5 (8%)		5 (7%)	3 (8%)	2 (7%)		4 (6%)	1 (4%)	3 (8%)	
Unknown	4 (3%)	1 (1%)	3 (5%)		4 (6%)	1 (3%)	3 (10%)		0 (0%)	0 (0%)	0 (0%)	
Molecular B cell status = B cell rich	59 (48%)	30 (48%)	29 (48%)	1	30 (48%)	15 (42%)	15 (56%)	0·402	29 (48%)	15 (56%)	14 (41%)	0·39
Synovial Pathotype				0·484				0·544				0.048
Fibroid	24 (18%)	13 (19%)	11 (17%)		11 (16%)	6 (15%)	5 (17%)		13 (20%)	7 (25%)	6 (16%)	
Lymphoid	74 (56%)	40 (60%)	34 (52%)		36 (53%)	21 (54%)	15 (52%)		38 (58%)	19 (68%)	19 (51%)	
Myeloid	31 (23%)	13 (19%)	18 (27%)		17 (25%)	11 (28%)	6 (21%)		14 (22%)	2 (7%)	12 (32%)	
Ungraded	4 (3%)	1 (1%)	3 (5%)		4 (6%)	1 (3%)	3 (10%)		0 (0%)	0 (0%)	0 (0%)	
Gender = Male	24 (18%)	8 (12%)	16 (24%)	0·105	16 (24%)	4 (10%)	12 (41%)	0·007	8 (12%)	4 (14%)	4 (11%)	0·967
Age, years	55·1 (13·3)	53·8 (13·3)	56·4 (13·2)	0·26	54·7 (13·7)	54·3 (13·9)	55·1 (13·5)	0·811	55·6 (13·0)	53·1 (12·5)	57·5 (13·1)	0·185
Disease Duration, years	9·0 [5·0, 19·0]	9·0 [4·5, 22·0]	9·0 [5·2, 17·8]	0·633	8·0 [4·0, 21·0]	10·0 [5·0, 22·5]	6·0 [4·0, 13·0]	0·146	10·0 [5·0, 18·0]	8·5 [4·0, 16·5]	10·0 [6·0, 19·0]	0·446
Ethnicity				0·111				0·884				0·038
African	13 (10%)	7 (10%)	6 (9%)		10 (15%)	6 (15%)	4 (14%)		3 (5%)	1 (4%)	2 (5%)	
Asian	9 (7%)	8 (12%)	1 (2%)		4 (6%)	3 (8%)	1 (3%)		5 (8%)	5 (18%)	0 (0%)	
Caucasian	104 (78%)	49 (73%)	55 (83%)		49 (72%)	27 (69%)	22 (76%)		55 (85%)	22 (79%)	33 (89%)	
Other	7 (5%)	3 (4%)	4 (6%)		5 (7%)	3 (8%)	2 (7%)		2 (3%)	0 (0%)	2 (5%)	
Clinical disease activity index (CDAI)	30·9 [21·7, 40·7]	28·9 [21·0, 39·9]	32·7 [23·2, 43·0]	0·431	31·0 [22·3, 41·1]	28·7 [20·0, 39·0]	33·2 [28·1, 46·8]	0·073	30·4 [21·7, 40·6]	31·8 [22·0, 40·5]	29·5 [21·3, 40·6]	0·582

Erythrocyte sedimentation rate (ESR), mm/h	32·0 [18·0, 46·0]	35·0 [18·5, 47·5]	28·0 [18·2, 43·0]	0·399	34·0 [17·8, 46·2]	38·0 [23·5, 49·5]	29·0 [17·0, 43·0]	0·262	28·0 [19·0, 45·0]	33·0 [16·5, 45·8]	27·0 [19·0, 45·0]	0·895
C-reactive protein (CRP), mg/L	11·0 [5·0, 23·0]	9·0 [4·0, 21·5]	11·5 [5·2, 27·8]	0·564	10·0 [5·0, 20·8]	8·0 [2·5, 16·0]	12·0 [5·0, 28·0]	0·138	15·0 [6·0, 29·0]	17·0 [8·0, 39·2]	11·0 [6·0, 27·0]	0·301
Rheumatoid factor (RF) OR Anti–citrullinated protein antibody (ACPA) positive	115 (86%)	58 (87%)	57 (86%)	1	59 (87%)	34 (87%)	25 (86%)	1	56 (86%)	24 (86%)	32 (86%)	1
Rheumatoid factor (RF) positive	96 (72%)	50 (75%)	46 (70%)	0·659	52 (76%)	30 (77%)	22 (76%)	1	44 (68%)	20 (71%)	24 (65%)	0·77
Anti–citrullinated protein antibody (ACPA) positive	104 (78%)	50 (75%)	54 (82%)	0·427	54 (79%)	31 (79%)	23 (79%)	1	50 (77%)	19 (68%)	31 (84%)	0·226
Haemoglobin, g/L	121·0 [110·0, 130·5]	118·0 [106·5, 130·5]	126·0 [115·5, 129·5]	0·134	120·0 [108·5, 131·5]	114·0 [101·0, 128·5]	128·0 [119·2, 135·2]	0·006	121·5 [111·0, 130·0]	122·0 [112·5, 138·8]	121·5 [109·8, 128·2]	0·452
Number of tender joints, 0-28	12·0 [6·0, 18·0]	12·0 [6·0, 16·5]	11·5 [7·0, 18·0]	0·69	11·0 [7·0, 18·2]	10·0 [6·0, 16·5]	13·0 [8·0, 21·0]	0·157	12·0 [6·0, 16·0]	12·0 [6·8, 16·2]	9·0 [6·0, 15·0]	0·457
Number of swollen joints, 0-28	6·0 [4·0, 10·0]	6·0 [3·0, 10·0]	6·0 [4·0, 12·0]	0·302	6·0 [4·0, 9·0]	5·0 [3·0, 8·0]	8·0 [5·0, 13·0]	0·023	6·0 [3·0, 11·0]	6·5 [3·8, 10·2]	6·0 [3·0, 12·0]	0·527
28 joint count Disease Activity Score (DAS-28), ESR	5·8 (1·2)	5·8 (1·2)	5·9 (1·2)	0·794	5·9 (1·2)	5·7 (1·2)	6·0 (1·2)	0·349	5·8 (1·3)	5·9 (1·3)	5·8 (1·3)	0·606
28 joint count Disease Activity Score (DAS-28), CRP	5·4 (1·2)	5·3 (1·2)	5·4 (1·2)	0·516	5·3 (1·1)	5·1 (1·1)	5·6 (1·1)	0·055	5·4 (1·2)	5·6 (1·2)	5·3 (1·2)	0·318
Ultrasound 12-max score (Power Doppler)	4·0 [0·5, 8·5]	2·0 [0·0, 7·5]	7·0 [4·0, 12·0]	0·012	4·0 [1·0, 8·0]	2·0 [0·5, 6·5]	6·0 [4·0, 9·8]	0·218	5·0 [0·2, 9·5]	1·5 [0·0, 8·0]	7·0 [4·5, 17·2]	0·02
Ultrasound 12-max score (Synovial Thickening)	17·5 (8·4)	15·9 (7·5)	19·4 (9·1)	0·09	17·9 (7·2)	17·2 (5·6)	18·7 (8·8)	0·525	17·0 (9·8)	14·4 (9·2)	20·3 (9·8)	0·103
van der Heijde modified Sharp score (SHSS), Total	8·8 [2·4, 41·5]	9·5 [2·4, 40·5]	8·8 [2·4, 44·8]	1	8·5 [2·5, 40·5]	8·5 [2·0, 40·5]	8·5 [2·9, 33·0]	0·843	10·5 [2·0, 53·2]	10·5 [2·5, 32·0]	11·0 [0·9, 57·0]	0·894
van der Heijde modified Sharp score (SHSS), Joint Space Narrowing	7·8 [1·0, 32·1]	8·0 [1·0, 30·2]	5·5 [1·8, 32·1]	0·98	5·0 [1·0, 30·0]	8·0 [1·0, 37·5]	3·0 [2·0, 16·1]	0·664	10·0 [1·0, 37·5]	9·5 [1·2, 28·2]	10·5 [0·8, 42·1]	0·653
van der Heijde modified Sharp score (SHSS), Erosion	1·8 [0·0, 11·0]	1·5 [0·0, 7·4]	3·5 [0·0, 11·8]	0·536	3·5 [0·0, 9·0]	1·5 [0·0, 8·5]	4·0 [0·5, 9·5]	0·512	0·5 [0·0, 12·5]	1·5 [0·0, 4·5]	0·5 [0·0, 17·9]	0·892
Previous Methotrexate use	133 (100%)	67 (100%)	66 (100%)		68 (100%)	39 (100%)	29 (100%)		65 (100%)	28 (100%)	37 (100%)	
Previous Prednisolone use	74 (56%)	39 (58%)	35 (53%)	0·67	35 (51%)	21 (54%)	14 (48%)	0·834	39 (60%)	18 (64%)	21 (57%)	0·72

Data are n (%), median [IQR], mean (SD). ECOG=Eastern Cooperative Oncology Group. BMI=body-mass index. CDAI=Clinical disease activity index. DAS28 =28 joint count disease activity score. CRP=C-reactive protein. ESR=erythrocyte sedimentation rate. All comparisons were two-sided; Student's T-test or Mann-Whitney-U test was used for continuous variables (based on normality of

distribution) and Chi-Squared test or Fisher’s exact test was used for categorical variables (based on low cell count occurrence, if at least one cell count in the 2xn contingency table was equal or less than 5, Fisher’s test was used). No adjustment for multiple comparison was performed.

Supplementary Table S7 | Antibodies used for immunofluorescence

	Species	Conjugation	Cat #	Conc. stock	Dilution
**Primary antibodies**					
**DKK3**	Rabbit	N/A	Sigma-Aldrich HPA011868	0.20 mg/ml	1:150
**CD45**	Mouse IgG1	N/A	Dako M0701	-	1:50
**CD90**	Rabbit	N/A	Abcam 133350	0.122 mg/ml	1:240
**Secondary antibodies and detection systems**					
**Dako Envision+ System-HRP,Labelled Polymer**	Anti-mouse	HRP	DAKO 4001	N/A	N/A
**Dako Envision+ System-HRP,Labelled Polymer**	Anti-rabbit	HRP	DAKO 4003	N/A	N/A
**AlexaFluor488 Tyramide reagent**	N/A	AF488	Invitrogen B40953	N/A	1:100
**AlexaFluor555 Tyramide reagent**	N/A	AF555	Invitrogen B40955	N/A	1:100
**AlexaFluor647 Tyramide reagent**	N/A	AF647	Invitrogen B40958	N/A	1:100
**DAPI,Dihydrochloride**	N/A	N/A	Cabiochem268298	N/A	1:1000

Name	Affiliation
Frances Humby	Guy's and St Thomas' NHS Foundation Trust, London, UK
Myles Lewis	Centre for Experimental Medicine and Rheumatology, Queen Mary University of London, London, UK
	Department of Rheumatology, Mile End Hospital, Barts Health NHS Trust, London, UK
Felice Rivellese	Centre for Experimental Medicine and Rheumatology, Queen Mary University of London, London, UK
	Department of Rheumatology, Mile End Hospital, Barts Health NHS Trust, London, UK
Alessandra Nerviani	Centre for Experimental Medicine and Rheumatology, Queen Mary University of London, London, UK
	Department of Rheumatology, Mile End Hospital, Barts Health NHS Trust, London, UK
Giovanni Giorli	Centre for Experimental Medicine and Rheumatology, Queen Mary University of London, London, UK
Michele Bombardieri	Centre for Experimental Medicine and Rheumatology, Queen Mary University of London, London, UK
	Department of Rheumatology, Mile End Hospital, Barts Health NHS Trust, London, UK
Georgina Thorborn	Centre for Experimental Medicine and Rheumatology, Queen Mary University of London, London, UK
Liliane Fossati-Jimack	Centre for Experimental Medicine and Rheumatology, Queen Mary University of London, London, UK
Costantino Pitzalis	Centre for Experimental Medicine and Rheumatology, Queen Mary University of London, London, UK
	Department of Rheumatology, Mile End Hospital, Barts Health NHS Trust, London, UK
Patrick Durez	Department of Rheumatology, Cliniques Universitaires Saint-Luc, Brussels, Belgium
	Institute of Experimental and Clinical Research (IREC), Université catholique de Louvain, Brussels, Belgium
Maya H Buch	Centre for Musculoskeletal Research, Division of Musculoskeletal & Dermatological Sciences, The University of Manchester, Manchester, UK
	NIHR Manchester Biomedical Research Centre, Manchester, UK
Hasan Rizvi	Department of Cellular Pathology, Barts Health NHS Trust, London, UK
	Institute of Health Sciences Education, Queen Mary, University of London, London, UK
Arti Mahto	Department of Rheumatology, Kings College Hospital NHS Foundation Trust, London, UK

Carlomaurizio Montecucco	Department of Rheumatology, Fondazione I.R.C.C.S. Policlinico San Matteo, University of Pavia, Pavia Italy
Bernard Lauwerys	Department of Rheumatology, Cliniques Universitaires Saint-Luc, Brussels, Belgium
	Institute of Experimental and Clinical Research (IREC), Université catholique de Louvain, Brussels, Belgium
Nora Ng	Rheumatology Department, Guy's and St Thomas' NHS Foundation Trust, London, UK
Pauline Ho	The Kellgren Centre for Rheumatology, Manchester Royal Infirmary, Manchester University NHS Foundation Trust, Manchester, UK
Vasco C Romão	Rheumatology Department, Hospital De Santa Maria, Centro Hospitalar Universitário Lisboa Norte, Lisbon, Portugal
	Rheumatology Research Unit, Instituto de Medicina Molecular João Lobo Antunes, Faculdade de Medicina, Universidade de Lisboa, Lisbon, Portugal
Patrick Verschueren	Skeletal Biology and Engineering (SBE) Research Centre, Department of Development and Regeneration, KU Leuven, Leuven, Belgium
	Division of Rheumatology, University Hospital Leuven, Leuven, Belgium
Stephen Kelly	Department of Rheumatology, Mile End Hospital, Barts Health NHS Trust, London, UK
Pier Paolo Sainaghi	Department of Rheumatology, University of Eastern Piedmont (UPO) and Maggiore della Carita Hospital, Novara, Italy
Nagui Gendi	Rheumatology Department, Basildon Hospital, Basildon, UK
Bhaskar Dasgupta	Rheumatology Department, Mid & South Essex University Hospitals NHS Foundation Trust, Southend University Hospital, Westcliff-on-Sea, UK
Alberto Cauli	Rheumatology Unit, Department of Medicine and Public Health, AOU and University of Cagliari, Monserrato, Italy
Piero Reynolds	Department of Rheumatology, Homerton University Hospital, London, UK
Juan D Cañete	Rheumatology Department, Hospital Clínic de Barcelona, Barcelona, Spain
	Institut d'Investigacions Biomèdiques August Pí I Sunyer (IDIBAPS), Barcelona, Spain
Robert Moots	Academic Rheumatology Unit, Aintree University Hospital, Liverpool, UK
	Faculty of Health, Social Care and Medicine, Edge Hill University, Ormskirk, UK

Peter C Taylor	Nuffield Department of Orthopaedics, Rheumatology and Musculoskeletal Sciences, Botnar Research Centre, University of Oxford, Oxford, UK
Chris J Edwards	NIHR Clinical Research Facility, University Hospital Southampton, Southampton, UK
	Faculty of Medicine, University of Southampton, Southampton, UK
John Isaacs	Translational and Clinical Research Institute, Newcastle University, Newcastle upon Tyne, UK
	Musculoskeletal Unit, Newcastle upon Tyne hospitals NHS Foundation Trust, Newcastle upon Tyne, UK
Peter Sasieni	King's Clinical Trials Unit, Kings College London, London, UK
Ernest Choy	CREATE Centre, Cardiff University, Cardiff, UK
	Department of Rheumatology, University Hospital of Wales, Cardiff, UK
Charlotte Thompson	Brighton and Sussex Medical School, University of Sussex, Brighton, UK
Serena Bugatti	Department of Rheumatology, Fondazione I.R.C.C.S. Policlinico San Matteo, University of Pavia, Pavia Italy
Mattia Bellan	Department of Rheumatology, University of Eastern Piedmont (UPO) and Maggiore della Carita Hospital, Novara, Italy
Mattia Congia	Rheumatology Unit, Department of Medicine and Public Health, AOU and University of Cagliari, Monserrato, Italy
Christopher Holroyd	Southampton General Hospital, University Hospital Southampton NHS Foundation Trust, Southampton, UK
Arthur Pratt	Newcastle University, Newcastle upon Tyne, UK
	Newcastle Hospitals NHS Trust, Newcastle upon Tyne, UK
João Eurico Cabral da Fonseca	Rheumatology Research Unit, Instituto de Medicina Molecular João Lobo Antunes, Faculdade de Medicina, Universidade de Lisboa, Lisbon, Portugal
Laura White	Cancer Research UK & UCL Cancer Trials Centre, University College London, London, UK
Louise Warren	Centre for Experimental Medicine and Rheumatology, Queen Mary University of London, London, UK
Joanna Peel	Centre for Experimental Medicine and Rheumatology, Queen Mary University of London, London, UK
Rebecca Hands	Centre for Experimental Medicine and Rheumatology, Queen Mary University of London, London, UK
Gaye Hadfield	Centre for Experimental Medicine and Rheumatology, Queen Mary University of London, London, UK
Julio Ramirez	Rheumatology Department, Hospital Clínic de Barcelona, Barcelona, Spain
Raquel Celis	Rheumatology Department, Hospital Clínic de Barcelona, Barcelona, Spain
