# Supplementary material for: Rituximab versus tocilizumab in rheumatoid arthritis: synovial biopsy-based biomarker analysis of the phase 4 R4RA randomized trial
Source: Nat Med. 2022 May 19;28(6):1256–68. doi: 10.1038/s41591-022-01789-0 (PMC9205785; doi:10.1038/s41591-022-01789-0)
Supplement: Supplementary file 2 — Reporting Summary [file 41591_2022_1789_MOESM2_ESM.pdf]

nature research

Corresponding author(s): Costantino Pitzalis

Last updated by author(s): Mar 18, 2022

Reporting Summary

Nature Research wishes to improve the reproducibility of the work that we publish. This form provides structure for consistency and transparency in reporting. For further information on Nature Research policies, see our Editorial Policies and the Editorial Policy Checklist.

Statistics

For all statistical analyses, confirm that the following items are present in the figure legend, table legend, main text, or Methods section.

n/a	Confirmed	
☐	☒	The exact sample size (*n*) for each experimental group/condition, given as a discrete number and unit of measurement
☐	☒	A statement on whether measurements were taken from distinct samples or whether the same sample was measured repeatedly
☐	☒	The statistical test(s) used AND whether they are one- or two-sided<br>*Only common tests should be described solely by name; describe more complex techniques in the Methods section.*
☐	☒	A description of all covariates tested
☐	☒	A description of any assumptions or corrections, such as tests of normality and adjustment for multiple comparisons
☐	☒	A full description of the statistical parameters including central tendency (e.g. means) or other basic estimates (e.g. regression coefficient) AND variation (e.g. standard deviation) or associated estimates of uncertainty (e.g. confidence intervals)
☐	☒	For null hypothesis testing, the test statistic (e.g. *F*, *t*, *r*) with confidence intervals, effect sizes, degrees of freedom and *P* value noted<br>*Give P values as exact values whenever suitable.*
☒	☐	For Bayesian analysis, information on the choice of priors and Markov chain Monte Carlo settings
☒	☐	For hierarchical and complex designs, identification of the appropriate level for tests and full reporting of outcomes
☐	☒	Estimates of effect sizes (e.g. Cohen's *d*, Pearson's *r*), indicating how they were calculated

Our web collection on statistics for biologists contains articles on many of the points above.

Software and code

Policy information about availability of computer code

Data collection	RNA-Seq: FastQC files were generated on an Illumina HiSeq instrument (performed by Genewiz (South Plainfield, NJ, USA)) and FASTQC files were returned. Transcript quantification using Salmon (version 0.13.1), index generation from Gencode release 29 transcriptome (gencode gene annotation v29) https://www.gencodegenes.org/human/release_29.html, Tximport (version 1.13.10), variance stabilizing transformation (VST) using the DESeq2 (version 1.25.9)<br>DSP:GeoMx WTA sequencing reads from NovaSeq6000 was compiled into FASTQ files corresponding to each ROI. FASTQ files were converted to Digital Count Conversion (DCC) files using the NanoString GeoMx NGS DnD (DnD 1.0) Pipeline.
Data analysis	Power calculations: edgeR (version 3.32.1), RNASeqPower (version 1.34.0)<br>RNA-Seq data and PCA analysis: DESeq2 (version 1.25.9), edgeR (version 3.32.1), limma (version 3.44.3), PCAtools (version 4.1)<br>Cluster analysis: edgeR (version 3.32.1), ComplexHeatmap (version 2.2.0), M3C (version 1.12.0), corrplot (version 0.90), DESeq2 (version 1.25.9), qvalue (version 2.22.0)<br>Differential expression and modular analysis: DESeq2 (version 1.25.9), edgeR (version 3.32.1), Limma (version 3.44.3), tmod (version 0.46.2), QuSAGE version 2.10.0, volcano3D (version1.0.3) https://cran.r-project.org/web/packages/volcano3D/index.html, qvalue (version 2.22.0), SummarizedExperiment (version 1.20.0)<br>Deconvolution: MCP counter (https://github.com/ebecht/MCPcounter), Seurat (version 3.2.0) Cross-over analysis: DESeq2 (version 1.25.9), volcano3D (version 1.0.3)<br>DSP analysis: DESeq2 R package (version 1.25.9) GeoMx NGS Pipeline (DND 1.0), qvalue (version 2.26.0), glmmSeq (version 0.1.0, https://cloud.r-project.org/web/packages/glmmSeq/index.html<br>), IHW (version 1.22)<br>Longitudinal mixed effects models analysis: lme4 (version 1.1-25), DESeq2 (version 1.25.9), Limma (version 3.44.3), car package (version 3.0-10), glmmSeq https://cloud.r-project.org/web/packages/glmmSeq/index.html<br>Longitudinal pathway analysis: cytoscape (version 3.7.2.), clueGO (version 2.5.5) GO/pathway repositories: BiologicalProcess-EBI-UniProt-GOA (11.02.2020), CellularComponent-EBI-UniProt-GOA (11.02.2020), ImmuneSystemProcess-EBI-UniProt-GOA (11.02.2020), MolecularFunction-EBI-UniProt-GOA (11.02.2020), KEGG (27.02.2019), REACTOME (27.02.2019)

Classifier models for predictions: caret (version 6.0-86), plotROC (version 2.2.1), glmnet (version 4.1-3), gbm (version 2.1.8), xgboost (version 1.5.0.2)
Web interface: R shiny server 1.5.16, R plotly 4.9.3, volcano3D (version 1.2.0)

For manuscripts utilizing custom algorithms or software that are central to the research but not yet described in published literature, software must be made available to editors and reviewers. We strongly encourage code deposition in a community repository (e.g. GitHub). See the Nature Research guidelines for submitting code & software for further information.

Data

Policy information about availability of data

All manuscripts must include a data availability statement. This statement should provide the following information, where applicable:

- Accession codes, unique identifiers, or web links for publicly available datasets
- A list of figures that have associated raw data
- A description of any restrictions on data availability

The datasets generated during and/or analysed during the current study are available on an interactive web interface that allows direct data exploration (https://r4ra.hpc.qmul.ac.uk/) A searchable interface is available to examine relationships between individual synovial gene transcript levels and histological and clinical parameters, and clinical response at 16 weeks. In addition, interactive versions of figures 3c, 5b and Extended Data Figure 5e and f allow users to click on individual genes to see their expression and search for genes of interest. The website was constructed using R shiny server 1.5.16 with interactive plots generated using R plotly 4.9.3.
The datasets can be downloaded from https://doi.org/10.6084/m9.figshare.19336679.
Other public datasets used for pathway analysis come from the Gene Ontology Annotation (GOA) database (BiologicalProcess-EBI-UniProt-GOA (11.02.2020), CellularComponent-EBI-UniProt-GOA (11.02.2020), ImmuneSystemProcess-EBI-UniProt-GOA (11.02.2020), MolecularFunction-EBI-UniProt-GOA (11.02.2020).), KEGG and Reactome.

Field-specific reporting

Please select the one below that is the best fit for your research. If you are not sure, read the appropriate sections before making your selection.

☒ Life sciences ☐ Behavioural & social sciences ☐ Ecological, evolutionary & environmental sciences

For a reference copy of the document with all sections, see nature.com/documents/nr-reporting-summary-flat.pdf

Life sciences study design

All studies must disclose on these points even when the disclosure is negative.

Sample size	According to the power calculation of the R4RA trial, a sample size of 82 B-cell-poor patients was assessed to provide 90% power to detect a 35% difference (assuming 55% response rate to Tocilizumab and 20% in Rituximab determined in previously conducted pilot study) in the proportion of patients who were deemed as responders by the primary endpoint (improvement in CDAI score of at least 50% at week 16). After estimating that 10% of biopsy samples would be ungradable and assuming a 5% dropout rate, a total of 160 patients would be required to recruit 82 patients who were B-cell poor.<br>n= 161 synovial samples were available at baseline and n=65 at 16 weeks. For molecular analyses (RNAsequencing), following quality control, as detailed below, n= 133 samples were available at baseline and 44 at 16 weeks.
Data exclusions	All the analyses presented herein were done in the intention-to-treat population. 164 patients were randomised but 3 patients did not receive the study drug, so were excluded from the intention-to-treat population. All baseline (n=161) and 16 weeks (n=65) synovial samples were sent for RNAsequencing. Following RNA-Seq quality control 36 samples were excluded due to poor mapping or RNA quality. Using unsupervised principal component analysis (PCA) and plotting the first 5 eigenvectors in pairs one outlier was identified and removed from further analysis. Thus 133 patients had RNA-Seq data available for subsequent analysis at baseline and 44 patients for the follow-up time point
Replication	For all patients, a minimum of 6 synovial samples were assessed by histology and a minimum of 6 samples were pooled for RNA extraction and RNA-sequencing, in order to limit sampling error and ensure that individual samples were representative of the whole synovial tissue, in line with EULAR and OMERACT consensus statement on minimal requirements for synovial biopsy analysis (https://doi.org/10.1186/s13075-018-1762-1). Semi-quantitative scores were performed on Immunohistochemical stainings of 3 cutting levels. To build classifier models for the prediction of treatment response (machine learning), due to the restricted sample size and the lack of a replication cohort, the dataset was split using 10x10-fold nested cross-validation. Sample sizes and replicates, where applicable, are indicated in the figure legends.
Randomization	At week 0, patients were randomly assigned (1:1) in block sizes of six and four to the rituximab group or the tocilizumab group stratified into four blocks according to histological classification of baseline synovial biopsy (B-cell poor, B-cell rich, germinal centre positive, or unknown) and by site (Queen Mary University London, London, UK vs all other sites) using an interactive web response system. More details on randomization are available in the publication reporting the primary trial results (Humby et al, Lancet 2021)
Blinding	Investigators and patients were blinded to the synovial pathotype, however the Ethics Committee advised against double-blinding the trial because it would be impractical and extremely inconvenient for patients. Since tocilizumab is given as monthly infusion, compared with rituximab, given every 6 months, blinding would have required all patients to have monthly infusions.

Reporting for specific materials, systems and methods

We require information from authors about some types of materials, experimental systems and methods used in many studies. Here, indicate whether each material, system or method listed is relevant to your study. If you are not sure if a list item applies to your research, read the appropriate section before selecting a response.

Materials & experimental systems

n/a	Involved in the study
☐	☒ Antibodies
☒	☐ Eukaryotic cell lines
☒	☐ Palaeontology and archaeology
☒	☐ Animals and other organisms
☐	☒ Human research participants
☐	☒ Clinical data
☒	☐ Dual use research of concern

Methods

n/a	Involved in the study
☒	☐ ChIP-seq
☒	☐ Flow cytometry
☒	☐ MRI-based neuroimaging

Antibodies

Antibodies used

For Immunohistochemistry, the following antibodies were used: CD79A (1:50 dilution, clone JCB117, catalogue number M7050, Lot number 41258342, 20057210, Agilent/Dako), CD3 (1:80 dilution, clone F7.238, catalogue number M7254, lot numbers 00086836, 20025164, Agilent/ Dako), CD20 (1:50 dilution, clone L26, catalogue number M0755, lot number 20023763 Agilent/Dako), CD68 (1:50 dilution, clone KP1, catalogue number M0814, lot numbers 00090015, 20025502, 20025503, 20025501, Agilent/Dako) and CD138 (1:50 dilution, clone MI15, catalogue number M7228, lot numbers 200033789, 20028635 Agilent/ Dako).

For multiplex immunofluorescence, the following antibodies have been used (more details in Supplementary table S7):
Anti-DKK3 Rabbit polycolonal Ab supplier Sigma-Aldrich Cat no:HPA011868, stock concentration 0.20mg/ml used in Dilution of 1:150.
Dako Envision System-HRP labelled polymer anti-rabbit cat no:4003(ready to use) applied as secondary antibody.
Alexafluor555 tyramide by Invitrogen cat no:B40955 diluted as 1:100 used as detection reagent.
CD45 Mouse IgG1 antibody by Dako, stock con:375mg/L, cat no:M0701 diluted as 1:50. Whereas, Dako Envision System-HRP, labelled polymer anti-mouse (ready to use) cat no:4001 used as secondary antibody.
Invitrogen Alexafluor647 cat no:B40958 used as in dilution 1:100.
CD90 Rabbit antibody by Abcam cat no:133350, stock concentration: 0.122mg/ml used in dilution as 1:240.Whereas, Dako Envision System-HRP, labelled polymer anti rabbit cat no:4003(ready to use) applied as secondary Ab.
Invitrogen Alexafluor488 cat no:B40953 used in dilution as 1:100.
DAPI, Dihydrochloride cat no:cabiochem268298 applied in dilution of 1:1000.

For GeoMx analysis, the following antibodies were used:
CD68-AF532 (clone KP-1, Novus, Cat#: NBP2-76575AF532, Lot MF-261), Dilution 1:100.
CD20-DL594 (clone IGEL/773, Novus, Cat# NBP2-47840DL594, Lot MF-550), Dilution 1:100.
CD3-AF647 (clone UMAB54, Origene, Cat#UM0000488F, Lot MF-659), Dilution 1:100.
Syto13 (NanoString, Cat# GMX-MORPH-NUC-12), Dilution 1:25.

Validation

All DAKO antibodies have been validated by the producer for in vitro diagnostic in human pathology, as detailed:
CD20 has been validated In normal lymphoid tissue, where it labels germinal centre cells, mantle zone lymphocytes, and scattered interfollicular lymphocytes, but not T cells, histiocytes and plasma cells. No labeling was observed in epidermis, sebaceous glands, hair follicles and eccrine glands in the skin, follicular epithelium in the thyroid, pneumocytes and bronchial epithelium of the lung, and a large number of other normal non-lymphoid tissues tested.
https://www.agilent.com/cs/library/packageinsert/public/SSM0755CEEFG_03.pdf

CD68 has been validated on normal peripheral blood and tissue resident monocytes, macrophages, Kupffer cells. Tissues tested include lung, liver, bone marrow, brain and kidney. Abnormal tissues tested included acute myeloid leukaemia cells and neoplasms of myeloid derivation, which showed strong and high levels (20/20) of labelling respectively. Negative controls included 100% of 22 T-cell lymphomas and 12 CD30+ anaplastic large-cell lymphomas were unlabelled. Some weak staining can be observed in FDCs in dermatopathic lymphoadenopathy and 1% plasma cell hyperplasias.
https://www.agilent.com/cs/library/packageinsert/public/SSM0814CEEFG_02.pdf

CD3 was validated on thymus, tonsil, lymph node resident cells, which showed strongly labelled cells in the medulla and cortex of the thymus and interfollicular areas of the other tissue types. Abnormal tissue testing included T-cell lymphomas and non-Hodgkin's lymphomas showing 41/52 cases and 100% of cases labelled, respectively. Negative control stains included 0/37 positive cases of different B-cell lymphomas.
https://www.agilent.com/cs/library/packageinsert/public/SSM7254CEEFG_02.pdf

CD138 staining was tested in bone marrow cells from multiple myeloma patients, all plasma cell types are labelled including reticular, polymorphous, asynchronous and basic plasma cells. Negative control staining included peripheral blood leucocytes from normal blood which showed <5% of cells stained positive.
https://www.agilent.com/cs/library/packageinsert/public/SSM7228CEEFG_02.pdf

CD79a was validated in peripheral and immature B-cell lines and showed no staining. However, staining is observed in B cells from embedded tissue sections. In normal tissue plasma cells are strongly labelled whilst in tonsillar tissue Germinal centre B-cells are highly labelled. In abnormal tissue 100% of 331 different B-cell neoplasms were labelled. As a negative control one study examined 98 different T-cell and non-lymphoid neoplasms and showed no positive staining. However, some precaution is needed as 2 separate studies showed positive staining in 10% of T-cell neoplasms/ T-lymphoblastic leukemia/lymphoma cases and a high level of staining in blast cells.

https://www.agilent.com/cs/library/packageinsert/public/SSM7050CEEFG_02.pdf

All antibodies have been further optimized for use in synovia by testing several dilutions and using isotype controls. The synovial CD20 staining/score has been also validated as described in Rivellese F, et al. Arthritis Rheumatol. 2020. (https://doi.org/10.1002/art.41184).

Antibodies used for immunofluorescence are commercially available and have been validated by the producer for use in immunofluorescence. In addition:
DKK3 has undergone enhanced validation by the Human Protein Atlas (HPA) project (https://www.proteinatlas.org/ENSG00000050165-DKK3/antibody).
CD90 has been used in synovia (Stephenson et al Nat Commun. 2018; 9: 791.).

Human research participants

Policy information about studies involving human research participants

Population characteristics	Patients aged 18 years or over, fulfilling 2010 ACR/EULAR classification criteria for Rheumatoid Arthritis who were eligible for treatment with rituximab therapy according to UK NICE guidelines, i.e. failing or intolerant to csDMARD therapy and at least one biologic therapy (excluding trial IMPs). Complete patient baseline characteristics are available in the manuscript describing the study results (Humby et al, Lancet 2021, DOI:https://doi.org/10.1016/S0140-6736(20)32341-2).<br>Characteristics tested as covariates in differential gene expression analysis were:<br>age: for all RNA-Seq patients in years 55.1 (standard deviation 13.3), rituximab treated RNA-Seq patients 54.7y (13.7 SD), tocilizumab treated RNA-Seq patients 55.6 (13.0 SD)<br>gender: in percentage of male 18% (all), 24% (RTX) and 12% (TOC)<br>ethnicity: for all RNA-Seq patients 10% African, 7% Asian, 78% Caucasian, 5% other; RTX 15% African, 6% Asian, 72% Caucasian, 7% other; TOC 5% African, 8% Asian, 85% Caucasian, 3% other
Recruitment	As above + inclusion/exclusion criteria, as detailed in the study protocol, available here: www.r4ra-nihr.whri.qmul.ac.uk/docs/r4ra_protocol_version_9_30.10.2017_clean.pdf. Patients were approached by their rheumatologist regarding participation in the trial during routine visits to outpatients clinics. Patients were given a patient information sheet and allowed sufficient time to discuss and consider their participation in the trial (at least 24 hours) prior to informed consent being taken. Participants did not receive any compensation, except for reimbursement of travel expenses.
Ethics oversight	MREC reference: 12/WA/0307 (https://www.hra.nhs.uk/planning-and-improving-research/application-summaries/research-summaries/r4-ra/). The ethics protocol has been approved by the following centres:<br>UK Ethics Committee<br>• Wales REC 3 (formerly "REC for Wales")<br>Local Ethics Committees in EU sites<br>• Comité d'Ethique Hospitalo-Facultaire<br>• Comissão de Ética para a Investigação Clínica (CEIC)<br>• Comitato Etico Interaziendale AOU "Maggiore della Carità" di Novara, ASL BI, ASL NO, ASL VCO<br>• Commissie Medische Ethiek UZ KU Leuven/Onderzoek<br>• Comité Ético de Investigación Clínica del Hospital Clínic de Barcelona<br>• Comitato Etico, Fondazione IRCCS Policlinico San Matteo<br>• Regione Autonoma della Sardegna Azienda Ospedaliero Universitaria di Cagliari Comitato Etico Indipendente

Note that full information on the approval of the study protocol must also be provided in the manuscript.

Clinical data

Policy information about clinical studies
All manuscripts should comply with the ICMJE guidelines for publication of clinical research and a completed CONSORT checklist must be included with all submissions.

Clinical trial registration	ISRCTN97443826 and EudraCT 2012-002535-28
Study protocol	www.r4ra-nihr.whri.qmul.ac.uk/docs/r4ra_protocol_version_9_30.10.2017_clean.pdf
Data collection	Clinical data was collected between Feb 28, 2013, and Jan 17, 2019 during study visits within the Rheumatology departments of participating sites. Participating sites were located at 19 hospitals in Europe (UK, Italy, Belgium, Portugal, and Spain). Please see the complete list of partecipating sites below:<br><br>• Mile End Hospital and Whipps Cross Hospital, Bart's Health NHS Trust, London, UK<br>• Cliniques Universitaires Saint Luc, Louvain, Belgium<br>• Santa Maria Hospital, Lisbon, Portugal<br>• Azienda ospedaliera Maggiore della Carità, Novara, Italy<br>• University Hospital of Wales, Cardiff and Vale University Health Board, Cardiff, UK<br>• Royal Victoria Infirmary, Newcastle upon Tyne Hospitals NHS Foundation Trust, Newcastle upon Tyne, UK<br>• Southampton General Hospital, University Hospital Southampton NHS Foundation Trust, Southampton, UK<br>• Basildon University Hospital, Mid and South Essex NHS Foundation Trust (formerly Basildon and Thurrock University Hospital NHS Foundation Trust), Basildon, UK<br>• Hospital Clínic de Barcelona, Barcelona, Spain<br>• Southend University Hospital, Mid and South Essex NHS Foundation Trust (formerly Southend University Hospital NHS Foundation Trust), Southend, UK

- Chapel Allerton Hospital, Leeds Teaching Hospitals NHS Trust, Leeds, UK
- Azienda Ospedaliero Universitaria di Cagliari, Cagliari, Italy
- Homerton University Hospital, Homerton University Hospital NHS Foundation Trust, London, UK
- Nuffield Orthopaedic Hospital, Oxford University Hospitals NHS Foundation Trust, Oxford, UK
- Aintree University Hospital, Aintree University Hospital NHS Foundation Trust, Liverpool, UK
- Manchester Royal Infirmary, Manchester University NHS Foundation Trust, Manchester, UK
- Guy's Hospital, Guy's and St Thomas' NHS Foundation Trust, London, UK
- Fondazione I.R.C.C.S. Policlinico San Matteo, Pavia, Italy
- Universitair Ziekenhuis Leuven, Leuven, Belgium

Outcomes

The primary outcome of the R4RA trial was defined as a binary outcome of treatment response using CDAI (Clinical disease activity Index) at 16 weeks after baseline. A responder was defined as CDAI improvement of greater than or equal to 50% from the baseline; a non-responder is defined as less than 50% improvement from baseline. The improvement is calculated as baseline CDAI (CDAI at week 0) minus CDAI at week 16. Secondary endpoints were defined using CDAI (CDAI MTR- CDAI improvement>50% and CDAI<10.1, CDAI<10.1) and DAS28 score (EULAR criteria, DAS28<3.2, DAS<2.6).
For more details on primary and secondary outcomes, please see the main publication (Humby et al, Lancet 2021, DOI:https://doi.org/10.1016/S0140-6736(20)32341-2)
